# Kombucha–Proteinoid Crystal Bioelectric Circuits

**DOI:** 10.1021/acsomega.4c07319

**Published:** 2024-10-28

**Authors:** Panagiotis Mougkogiannis, Anna Nikolaidou, Andrew Adamatzky

**Affiliations:** † Unconventional Computing Laboratory, University of the West of England, Coldharbour Ln, Stoke Gifford, Bristol BS16 1QY, U.K.; ‡ School of Architecture and Environment, University of the West of England, Coldharbour Ln, Stoke Gifford, Bristol BS16 1QY, U.K.

## Abstract

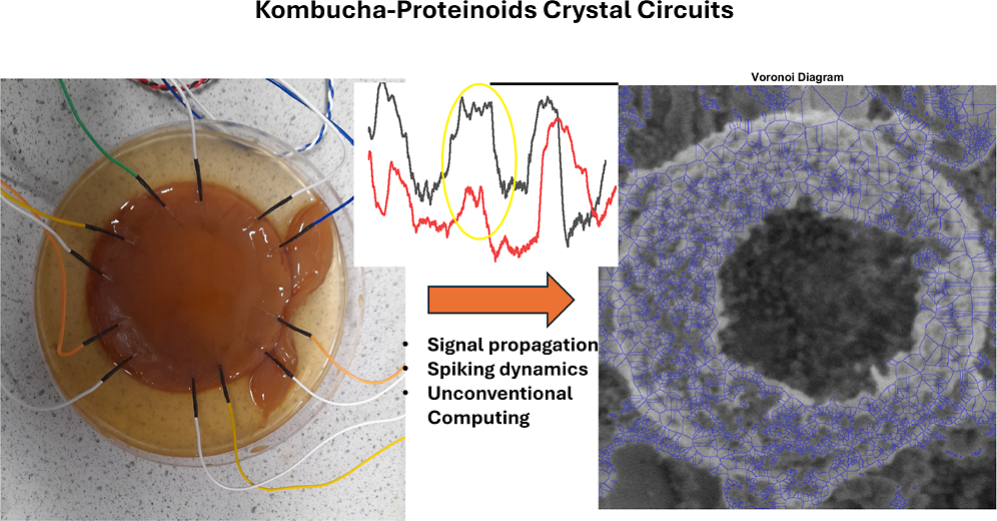

We propose “kombucha–proteinoid crystal bioelectric circuits” as a sustainable bio-computing platform. These circuits are hybrid biological-inorganic devices that utilize crystal growth dynamics as the physical substrate to convert information. Microfluidic prototypes couple custom-synthesized thermal proteinoids within kombucha cellulose matrices and metastable calcium carbonate solutions. This bio-mineral configuration examines if precision modulation of crystal growth rates could instantiate reconfigurable logic gates for unconventional computing applications. Programming organic acid secretions allows for the adjustment of biotic-mineral polarity, thereby establishing microbial-synthetic pairings that consistently regulate the crystal growth rate of calcite deposition. By coordinating intrinsic physicochemical phenomena, accrued mineral densities literally crystallize additive/multiplicative operations via Boolean AND/OR logics. An additional way to generate structured logics similar of neural assemblies is by chaining modular crystallizer units. Proteinoid-guided carbonate crystallization may prove to be a viable material platform for unconventional computing-green, self-organizing, scalable architectures grown directly from solution-pending definitive affirmation of proof-of-concept.

## 1 Introduction

Unconventional computing^1−5^ investigates information processing techniques that manifest intrinsically across many physical, chemical, and biological substrates.^6−10^ While there are numerous theoretical prototypes that demonstrate the possibility for biological computing systems^11−15^ that exploit chaotic dynamics, self-organization, and collective behaviors found in nature,^16−19,21,22,29^ there are few working experimental demonstrations that convert abstract concepts to experimental laboratory prototypes.^23−27^ However, any tangible materials system that demonstrates previously unknown aspects of morphological computation and dynamical inference enhances understanding of how “real-world” physics naturally computes in the absence of human intervention.^28,29,31,39^

Recent progress in bioinspired computation is facilitating the development of advanced biological computing platforms that utilize the natural information processing capacities of living systems at different levels.^20,30^ DNA,^11^ RNA,^32^ and proteins^33^ are being manipulated at the molecular level to create logic gates, circuits, and neural networks capable of executing pattern recognition, optimization, and other computing tasks. At the cellular level, scientists are creating models of gene regulatory networks and signaling pathways in order to comprehend the complex computations that occur during decision-making, memory formation, and adaptability.^13,14^ Membrane computing, a theoretical framework, offers novel paradigms for parallel and distributed computing in biological systems.^34^ Multicellular systems and organisms possess impressive computational skills that result from the coordinated actions of numerous interacting components. Slime molds possess the ability to tackle complex optimization challenges, such as identifying the most effective routes in mazes^35^ or designing optimized transportation networks.^36^ Ant colonies also exhibit cognitive hunting behaviors that have served as inspiration for highly effective optimization systems.^37^ Current research is beginning to utilize similar capacities in artificial biological systems, such as employing gene circuits in bacteria to carry out associative learning.^38^ Synthetic biology methods, such as genetic circuit design and directed evolution,^39^ enable systematic engineering of biological systems to perform computing tasks. Nevertheless, our understanding of biological information-processing is still restricted in comparison to silicon-based computing.^40^ Gaining a deeper understanding of how evolution has fine-tuned biological systems to efficiently carry out computations in noisy and uncertain situations might provide valuable insights for developing new bioinspired computing platforms and enhancing the efficiency of artificial computing systems.^41^ To do this, it will be crucial to combine theoretical frameworks from computer science, control theory, and statistical physics with experimental methods from synthetic biology and neuroscience.^42^ Incorporating the self-organization, adaptability, and efficiency of biological systems has the potential to bring about significant advancements in computing technology.^43^ This study presents a novel experimental configuration that combines biological and inorganic elements. It uses directed calcium carbonate crystal nucleation together with programmable kombucha–proteinoid neuromorphic circuits to control the pathways of crystal growth.^44,45^ Studying the unique ability of this living material to process signals offers valuable knowledge on the architecture of “smart matter.” This matter will transition from disordered solutions into interactive and adaptable electronics, using its acute sensitivity to dynamic changes.^46^ Chemical systems with reaction-diffusion interactions provide capable experimental substrates for geometry-solving searches for stable concentration patterns.^47−53^ Groups have gone on to build reaction-diffusion prototypes that perform optimization tasks and even functional logic gates.^54−57^ Beyond chemistry, slime mold organisms demonstrate complicated optimization behaviors that are analogous to conventional computing circuits establishing decision maps.^50,58,59^ Researchers implant living mold onto microfluidic lab-on-chip devices to take advantage of intrinsic parallel search algorithms honed via evolution.^60−63^ Furthermore, pioneering studies in quantum computing target defect-tolerant platforms that use collective solid-state spin entanglement to achieve machine learning goals.^64^ These selected instances provide views into natural computing frontiers where dynamics long considered as irrelevant noise instead execute sophisticated transformations relevant for electronic information systems trying to match nature’s computational efficiencies. We provide a hybrid biological-inorganic experimental setup that employs guided calcium carbonate crystal formation with programmable kombucha–proteinoid cineuromorphic circuits to direct propagation paths.^44^ Exploring this living material’s unusual signal processing capacity promises new insights into constructed “smart matter,” which will bridge from disordered precursor solutions into interactive, adaptive electronics formed straight from acute dynamical sensitivities.^46^

Researchers are investigating unconventional materials and architectures in an effort to find new computer paradigms that may be able to overcome the drawbacks of standard silicon-based systems.^65^ Given these circumstances, the use of hybrid bioabiotic composite materials in computing has become increasingly attractive.^66^ These materials offer special benefits such self-organization, flexibility, and energy efficiency. We have chosen a kombucha–proteinoid crystal hybrid system that takes advantage of the complementary characteristics of both living and nonliving elements. The proteinoid microspheres offer programmable excitability and information processing capabilities,^67^ while the self-assembling, conductive kombucha biofilm has inherent electrochemical features.^68^ Combining these two enables the development of bioelectric circuits capable of displaying complex, emergent behaviors similar to those of biological neural networks.^69^ Benefits from incorporating live cells into the composite material include the ability for computing functions to evolve, self-healing, and environmental responsiveness.^70^ It does, however, also come with difficulties in terms of long-term viability, stability, and reproducibility.^71^ Although the initial research we conducted has yielded encouraging outcomes for computational efficiency, the composite material is aging and will eventually display changes in its electrical characteristics and structural integrity.^72^ Although this aging process may have drawbacks, it also presents fascinating opportunities to investigate the evolution of computation in living systems.^73^

## 2 Materials and Methods

### 2.1 Kombucha–Proteinoid Neuromorphic Circuits Synthesis

**Figure 1:**
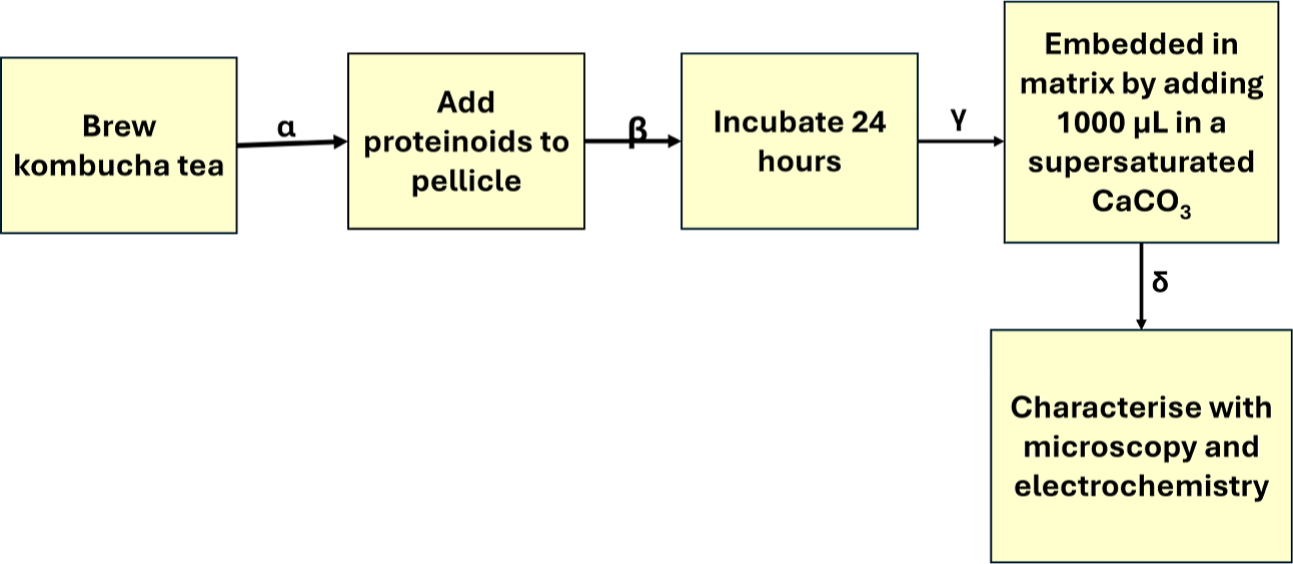
Synthesis of kombucha–proteinoid complexes. The image displays the critical processes in the fabrication of a composite consisting of kombucha cellulose matrix and proteinoid microspheres. Initially, the kombucha biofilm is grown through tea and sugar fermentation. Following that, polymerized proteinoid spheres are injected and surface-attached into the cellulose pellicle, allowing diffusion into the conductive hydrogel scaffold. After that, the composite is incubated to allow the proteinoids to fully infiltrate the kombucha matrix. The neuromorphic circuits is placed in a CaCO_3_ supersaturated solution to mimic biome conditions for characterization, and measurements are obtained using microscopy and electrochemistry. Tuning growth parameters such as culture duration, material ratios, and assembly kinetics allows for more exact integration of the components in this biotic–abiotic hybrid system. The modular biofabrication approach combines kombucha’s rapid scaffold synthesis with proteinoids’ programmable excitability to create smart, living electronic materials with emergent features. The arrows labeled α through δ indicate the sequence of fabrication steps.

Kombucha–proteinoid complexes were synthesized by integrating thermal polymerized proteinoids within kombucha cellulose pellicles as depicted in Figure 1. The proteinoids were produced using previously established techniques.^74^ Kombucha films were first cultured by steeping tea and sugar in boiled water, inoculating with a symbiotic SCOBY mat, and incubating at 20–23 °C in darkness. Once a cellulosic biofilm formed after 10–14 days, proteinoids solutions were introduced by injection into the mat and surface application. The composite was returned to the incubator for 24 h to enable proteinoid diffusion and attachment.

**Figure 2:**
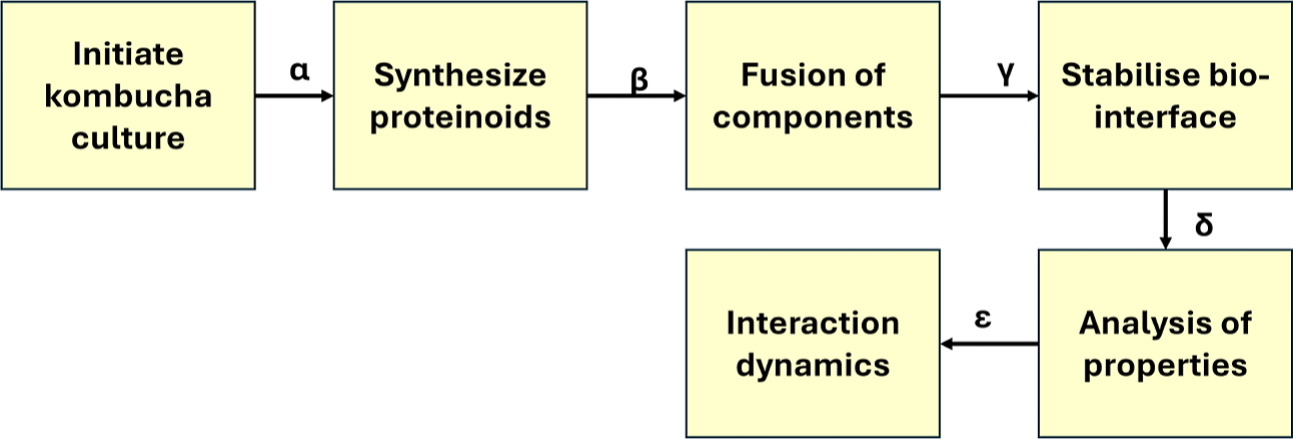
Dynamic synthesis and analysis of kombucha–proteinoid biointerface. This figure illustrates the cyclical process of creating and analyzing the biocomputing interface. The process begins with the initiation of the kombucha culture, followed by the synthesis of proteinoids. These components are then fused to form the biointerface, which is subsequently stabilized for interaction dynamics. The final stage involves a thorough analysis of the interface’s properties, leading back to the initiation stage to refine the process. The sequence of steps from α to ϵ represents the iterative nature of the experimental design, emphasizing the continuous interaction and development of the biocomputing platform.

The cyclical process of creating and analyzing the kombucha–proteinoid biocomputing interface is the primary focus of Figure 2. It highlights the iterative nature of the experimental design, with the biocomputing platform’s continuous interaction and development represented by the steps from α to ζ. The procedure entails the following steps: the initiation of the kombucha culture, the synthesis of proteinoids, the fusion of these components to create the biointerface, the stabilization of the interface for interaction dynamics, and the analysis of the interface’s properties prior to the process refinement. Continuous refinement and optimization are crucial in the development of biocomputing interfaces, as illustrated in Figure 2, which illustrates the dynamic synthesis and analysis process. Researchers can gain valuable insights into the properties and performance of the kombucha–proteinoid biointerface by iterating through the stages of initiation, synthesis, fusion, stabilization, and analysis. This enables them to make informed decisions on how to improve the system. Conversely, the fabrication process requires precision and complexity, as demonstrated in Figure 1, through the synthesis of kombucha–proteinoid complexes. The order of steps, which includes the growth of the kombucha biofilm and collection of measurements using microscopy and electrochemistry, illustrates the complexity of incorporating abiotic and biotic components into a functional hybrid system. The flexible biofabrication approach, which integrates the programmable excitability of proteinoids with the rapid scaffold synthesis of kombucha, has the potential to develop intelligent, living electronic materials with emergent characteristics.

The kombucha matrix provides a rapidly growing, tunable scaffold, while proteinoids confer tailored signaling dynamics. Resulting composites exhibit periodic electrical spikes reminiscent of neuronal action potentials. By combining kombucha’s structural qualities with proteinoids’ excitability, adaptable bioelectronic materials with emergent capabilities are produced.

Optimizing factors influencing integration like culture duration, materials ratios, and assembly kinetics will enable enhanced neuromorphic circuits synthesis. As shown in Figure 1, kombucha cultivation precedes introduction of programmed proteinoids, allowing precise engineering of living electronic composites.

The kombucha growth medium consisted of a calcium carbonate supersaturated solution containing 4.75 × 10^–3^ M initial Ca^2+^ concentration and a 1:1 calcium ion to carbonate ion molar ratio for seedless experiments and 3 × 10^–3^ M Ca^2+^ for seeded experiments at an ionic strength of 0.06 mol/L. The solution was prepared using Ca(NO_3_)_2_·4H_2_O, KNO_3_, and NaHCO_3_ reagents purchased from Sigma-Aldrich and used as received.

Scanning electron microscopy (SEM) was used to examine the structures of proteinoids and kombucha, employing FEI Quanta 650 equipment.

### 2.2 Electrical Measurements

The proteinoid microspheres were immersed in the kombucha solution described above. Platinum–iridium electrodes (diameter 0.1 mm) separated by a distance of 10 mm were embedded in the sample and connected to an external circuit. Input signals were provided by a RIGOL DG4162 function generator, configured to supply a 16.7 kHz sinusoidal wave with 5.00 V peak-to-peak amplitude. The corresponding electrical output signals were recorded using a Pico Technology Picoscope 4000 Series data recorder.

Impedance and capacitance measurements of the kombucha–proteinoid crystal composites were performed using a Zimmer Peacock potentiostat (ANAPOT model). Calcium ion (Ca^2+^) concentrations within the kombucha films and crystallization solutions were quantified utilizing specialized calcium ion sensors (ZPS CAL-000-00080) in conjunction with the potentiostat system. pH measurements were carried out using a combined Ag/AgCl and glass electrode (EDT directION Limited) with data capture via a Medgetech pHTemp2000 pH/temperature recorder.

**Figure 3:**
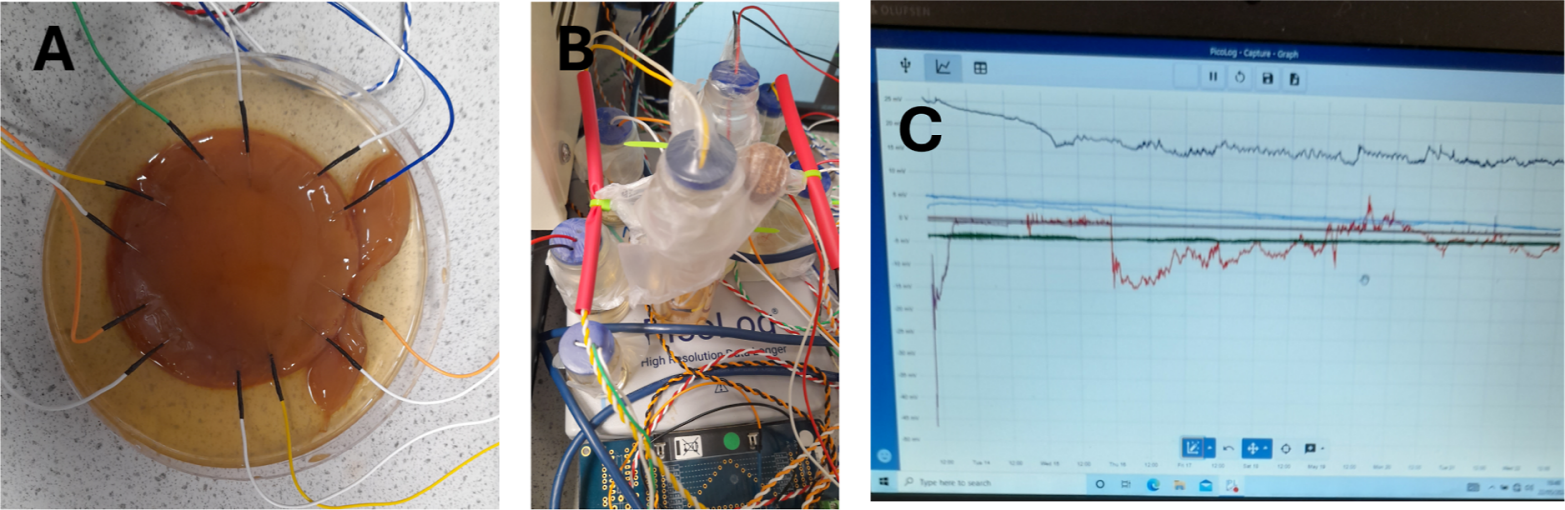
Experimental setup and data acquisition. (A) Platinum–iridium electrodes inserted in kombucha culture. (B) Electrodes inserted in a solution of kombucha and proteinoids. (C) Interface of the PicoLog software used for identifying and analyzing spiking dynamics from the recorded signals.

Figure 3 shows the experimental setup used to acquire electrical signal data from the kombucha cultures and kombucha–proteinoid solutions. Platinum–iridium electrodes were inserted into the kombucha culture (Figure 3A) and kombucha–proteinoid solution (Figure 3B) to record electrical activity. The recorded signals were then analyzed using the PicoLog software (Figure 3C) to identify spiking dynamics.

## 3 Results

### 3.1 Imaging Bio-Crystal Circuit Architectures

**Figure 4:**
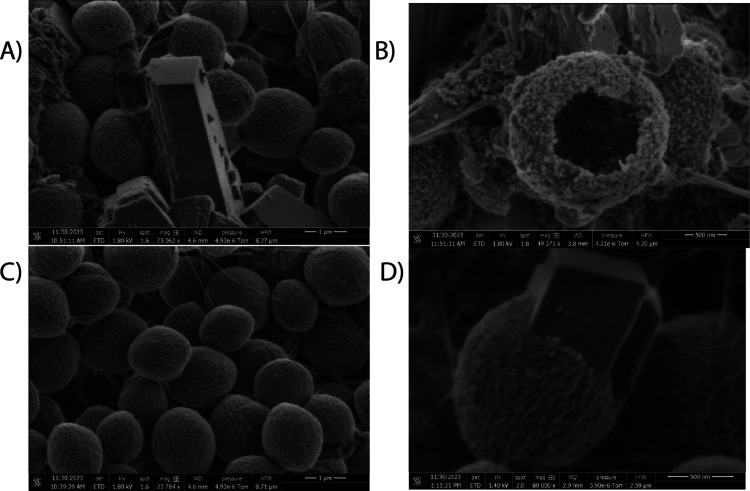
Directed crystallization trajectories demonstrating guided self-assembly of adaptable bio-mineral structures across preparation variants. (A) Kombucha mats (300 mg) generate polymorphic 1 μm mosaics of calcite and vaterite distinguished by optical distortion at corresponding initial calcium carbonate concentrations (3 × 10^–3^ M Ca^2+^M). (B) The addition of synthetic thermal proteins (1000 μL) results in hollow proteinoid spheres interconnected by branching mineral deposits; precursor calcium levels (4.75 × 10^–3^ M Ca^2+^M) define the resulting circuit nanostructure dimensions (500 nm). (C) Electron microscopy (23,784× magnification) displays composite 1 μm proteinoid microspheres with interior compartmentalizations, emphasizing hierarchical organization from molecular to ensemble strata. Finally, even pure spontaneous precipitations embed trace biological fingerprints (1000 μm proteinoids–kombucha) within layered vaterite–calcitic minerals as hints to temporary environments during encoded crystallization stages (D). The controlled blending of bespoke proteinoids with spontaneous self-assembling calcium carbonate creates hybrid-bioelectronic materials that combine microbial capabilities with planned modularity.

Scanning electron micrographs reveal detailed patterns and uneven forms within the crystalline bio-electric circuits, which are created by the complex branching of calcium carbonate lattices under the guidance of microbial and peptide populations. The composite photos demonstrate the blending of delicate hydrogels containing cells with imprints from harder mineral deposits. Additional amplification reveals minuscule bacteria and isolated proteinoids scattered throughout the common extracellular space. Therefore, the combined interactions at both the microscopic and macroscopic levels work together to organize the manufacturing process in order to achieve specific capabilities. Elemental mapping reveals the distribution patterns of carbonate, calcium, and organic materials, exposing chemical gradients that are likely responsible for the formation of highly reactive conduction zones. Meanwhile, electron backscatter diffraction reveals significant differences in the crystalline structure of various parts, which explains the apparent interruptions in the signal due to mismatched or blocked paths of propagation. The layered internal architecture that determines the exterior electronic characteristics is revealed by a comprehensive examination using microscopy. The ongoing endeavors to simulate the process of biotic-mineral self-assembly are focused on comprehending the intricate dynamics that govern the creation of functional crystal computers in many environments, including microbial, proteinoid, and crystal realms. The directed crystallization trajectories depicted in Figure 4 illustrate the guided self-assembly of adaptable biomineral structures across a variety of crystal growth methods. Figure 4A illustrates that kombucha mats (300 mg) produce polymorphic 1 μm mosaics of calcite and vaterite, which can be readily recognized by their unique morphology at corresponding initial calcium carbonate concentrations (3 × 10^–3^ M Ca^2+^M). This finding confirms the kombucha mats’ capacity to stimulate the development of complex mineral structures with unique morphologies and compositions, based upon the initial calcium carbonate concentration. The formation of hollow proteinoid spheres interconnected by branching mineral deposits is the result of the addition of synthetic thermal proteins (1000 μL), as illustrated in Figure 4B. The resulting crystal nanostructure dimensions (500 nm) are defined by the precursor calcium ions levels (4.75 × 10^–3^ M Ca^2+^M), suggesting that the concentration of calcium ions is a critical factor in determining the size and morphology of the proteinoid–mineral hybrid structures. Figure 4C illustrates the hierarchical organization of these structures from molecular to ensemble layers through electron microscopy images (23,784× magnification). The images depict composite 1 μm proteinoid microspheres with interior compartmentalizations. Finally, Figure 4D illustrates the fact that pure spontaneous precipitations incorporate trace biological fingerprints (1000 μm proteinoids–kombucha) into layered vaterite-calcitic minerals. These fingerprints serve as indicators of temporary environments during encoded crystallization phases. The results of these studies indicate that the intentional combination of tailored proteinoids with spontaneously self-assembling calcium carbonate results in hybrid-bioelectronic materials that integrate planned modularity with microbial capabilities.

### 3.2 Biologically-Directed Mineralization as Intrinsically Boolean Gates

This section examines the intricate and efficient formation of interconnected networks of bio-mineral residuals, which accumulate and flow together in a logical and hydrodynamically cohesive manner. Operationally, we investigate how mildly acidic secretions adjust the saturation ratios of precursor suspensions, which in turn control the growth of calcium carbonate crystals. Significant advancements arise: primarily, the analysis of neural spike train representations conveyed by pH or pCa time series inputs goes beyond traditional Boolean logics and instead focuses on functionally full axiom bases that utilize adaptive, state-morphing spatiotemporal mappings.

**Figure 5:**
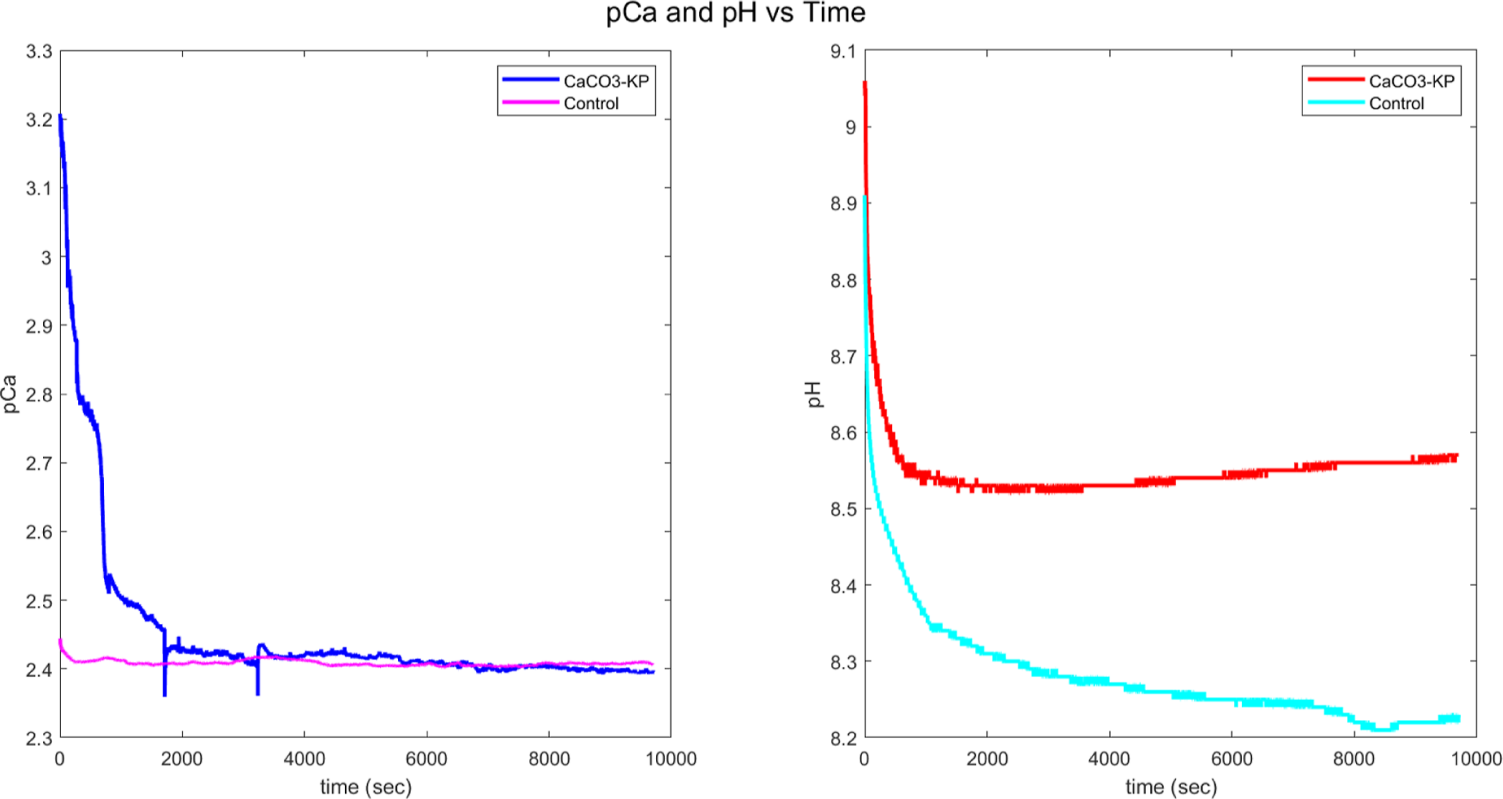
Graph displays how the acidity of the solution changes over time for kombucha–proteinoid calcium carbonate composites (CaCO_3_–KP) and a control solution of calcium carbonate (CaCO_3_). These values provide insight into the growth patterns influenced by the secretion of kombucha. The CaCO_3_–KP composite releases higher concentrations of acid, with a mean pCa of −0.008, stimulating the deposition of carbonate, which is essential for logical operations. In contrast, the control solution without proteinoids remains more alkaline with a mean pH of 8.282 and reduced saturation of carbonate. The greater acidification of the kombucha–proteinoid solution with average pH 8.551 indirectly reflects the activity of microbial sample, which influences the formation of computational structures by adjusting the saturation ratios. Even slight changes in pH have a significant impact on the crystallization pathways, particularly when dealing with interconnected protocell collectives.

As shown in Figure 5, acidic secretions from co-cultured kombucha–proteinoids provide internal tuning signals that direct structural assembly toward implementing logic processing. The spatial organization of kombucha–proteinoids, a group of bacteria that are co-cultured together, is influenced by the release of acidic molecules. These chemicals function as tuning signals that guide the construction of the structures in order to carry out logic processing. Through the measurement of pCa (calcium acidity) and pH levels at different time intervals, we can determine that certain conditions promote the formation of calcite, which is essential for the crystallization of logic gates. The kombucha–proteinoid sample exhibits an average pCa of 2.452 with stimulation of crystal accumulation compared to the basic control lacking synthetic networks. This increased proton concentration forms metastable phases, with pH dropping from an initial 8.551 to reflect proteinoid regulation of carbonate morphogenesis. This suggests that when the acidity exceeds a specific threshold, there is a faster build-up of crystals compared to a control solution that does not contain the synthetic network. The heightened concentration of protons triggers the formation of metastable phases. Furthermore, the pH of the sample decreases from an initial value of 8.551 to 8.520 during a period of 1526 s. The fluctuations in the internal environment of the kombucha–proteinoids indicate the participation of proteinoids that control the formation of carbonate structures. The recorded pCa and pH values can be interpreted as input signals in a system where their interactions and dynamic changes resemble some characteristics of neuromorphic computation, potentially offering insights into decision-making processes at the cellular level. When these values intersect predetermined thresholds, they merge to perform Boolean operations that can be interpreted from the collected concentrations of minerals. By establishing a connection between sensory stimuli and the regulation of output formation, we facilitate a process where crystal computer composites are autonomously formed in response to the environment. This technique utilizes the coordinating principles of microbial systems, in which local interactions propagate to induce global material transformations. This establishes a persuasive framework for designing “smart” matter, in which the material can adjust and react to its surroundings by following predetermined biological signals.

The collected mineral dynamics provide insight into the variety of logic gates that govern the coordinated crystallization of the kombucha–proteinoid networks. The ability to discern crucial bio-signals of extracellular acidity permits the execution of Boolean logic operations, such as AND
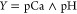
1and OR expressions
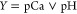
2where ∧ and ∨ represent the logic symbols. Even negated gates like NAND and NOR are exhibited through orchestrating accumulation events.

XOR gate can be also implemented
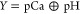
3where ⊕ represents XOR. Bitwise inversions also manifest through proteinoid inhibition of crystallization as the NOT gates
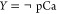
4
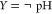
5

Combining NOT expressions enables set theoretic operations like NAND
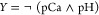
6and NOR
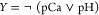
7

Finally, a XNOR response emerges from bi-conditional crystallization
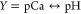
8

Ensemble computations exhibit a significantly broader spectrum of logical modalities, extending beyond basic growth to encompass the complexity of real-time processing. In effect, the living hybrid systems execute parallel biofilm-mediated computations spanning from the molecular to the population scales, constituting a decentralised crystal computer constructed entirely of common microbial life.

### 3.3 Capacitance Metrics of Crystal Bioelectronic Computers

Controlling crystal growth enables the customization of electrical properties in calcium carbonate supersaturated solutions. Kombucha contributes to the formation of intricate tubule structures, resulting in a significant capacitance of 40,389 nF due to the accumulation of carbonates. This capacitance value is equivalent to approximately 40.4 μF. To provide a more comparable metric, we can calculate the specific capacitance. Given the sample dimensions (diameter of 7 cm and thickness of 4.2 mm), we can express the specific capacitance in various ways.

- Per unit area: 10.5 μF/cm^2^ (based on the circular surface area of 38.5 cm^2^).
- Per unit volume: 25.0 μF/cm^3^ (based on the volume of 16.16 cm^3^).

Proteinoids restrict the formation of deposits, resulting in the creation of more resilient 78 × 10^–9^ structures instead. Different arrangements bring together bio-electronic components according to specific requirements—weaving crystalline logic gates or wires while carefully coordinating precursor conditions.^44^

**Figure 6:**
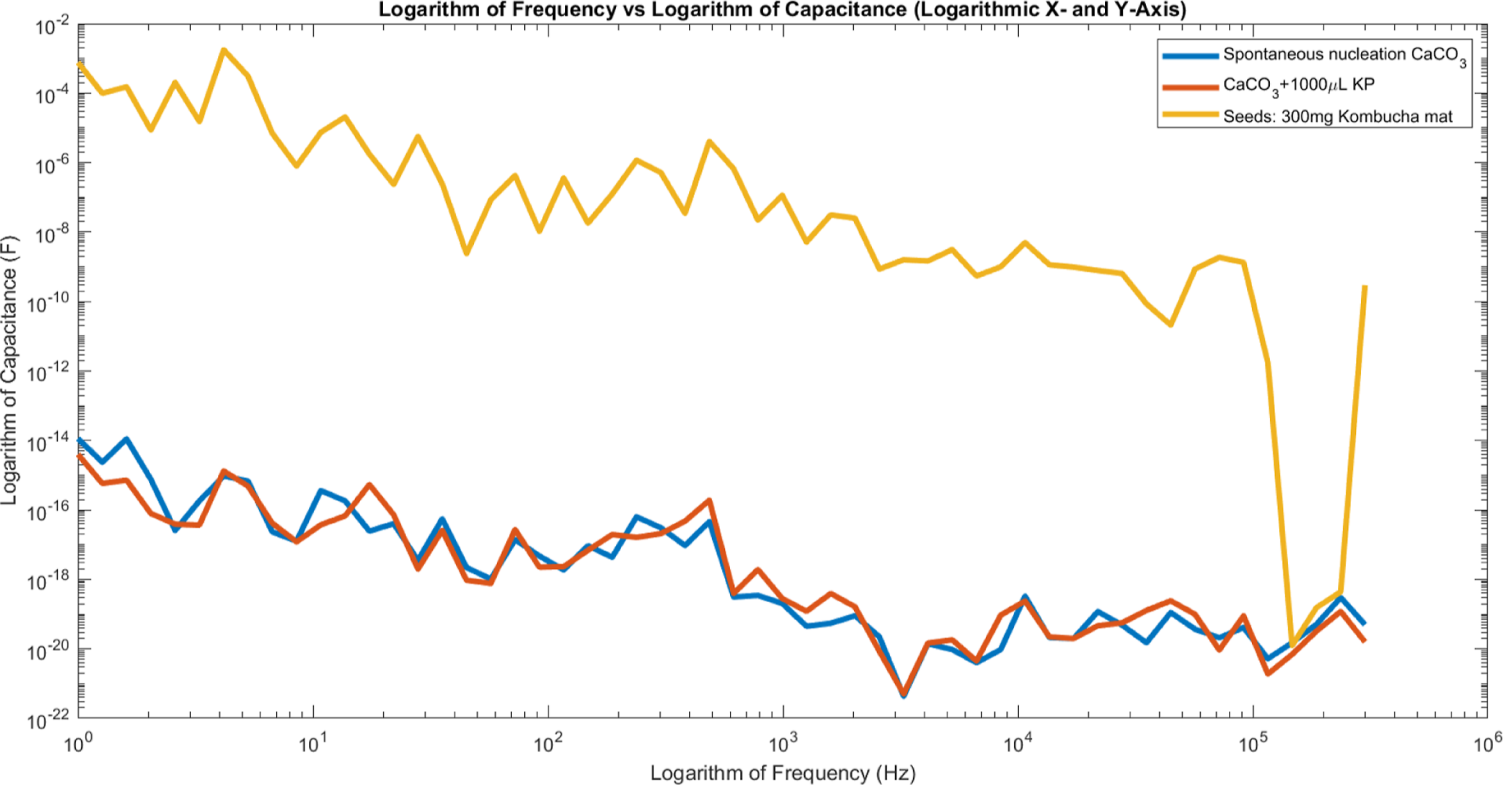
Illustration showcasing the variations in capacitive tuning throughout different calcium carbonate crystal growth methods. The *x*-axis displays the logarithm of frequency (Hz), representing a wide range of measurement frequencies from 1 Hz to 1 MHz on a logarithmic scale. This allows for the observation of capacitive behavior across several orders of magnitude in frequency, capturing both low-frequency and high-frequency responses of the systems. The *y*-axis shows the logarithm of capacitance (*F*), enabling comparison of capacitance values spanning many orders of magnitude. Control samples without any additives show an extremely low capacitance of 78 × 10^–9^ nF, while fabrics infused with kombucha produce significantly higher readings of 40,389 nF due to interconnected tubule structures. Proteinoid composites have a mean capacitance of −72 × 10^–9^ nF due to the hindered structural networking caused by moderated deposition. Small secretions have significant effects on global circuit architecture by guiding localized transformations-the core of intricate crystallization cascades that are responsive to initial disturbances. Metrics can be compared to highlight the significant impact of biological co-factors on capacitive solid-state electronics. These co-factors operate through fluid-phase biogeochemical ordering principles, which have the ability to shape the local environment and extend their influence to larger mineralizing matrices.

Figure 6 depicts the differences in capacitive tuning resulting from various techniques used for calcium carbonate crystal growth. The control samples, which did not have any additives, exhibited an extremely low capacitance of 78 × 10^–9^ nF. It appears that the crystal growth process has minimal impact on the material’s capacitive properties. On the other hand, fabrics that have been infused with kombucha, a fermented tea, showed much higher capacitance readings of 40,389 nF. The significant rise in capacitance can be credited to the interconnected tubule structures that develop within the crystal matrix, thereby improving the material’s electrical conductivity. However, the proteinoid composites showed an average capacitance of −72 × 10^–9^ nF. In some semiconductor devices, the capacitance can show complex behavior, such as negative values. As discussed in,^75^ negative capacitance occurs when the time-derivative of the transient current in response to a minor voltage step is nonmonotonic or positive-valued. This phenomenon has been theoretically predicted and experimentally confirmed in quantum well infrared photodetectors. The negative capacitance effect is attributed to the nonequilibrium transient injection from the emitter, which is a result of the injection barrier’s properties and the inertia of the quantum well recharging. This value for negative capacitance indicates a reduction in the overall capacitive properties. There is a theory suggesting that the restricted connectivity of proteinoid materials is a contributing factor in this situation. The deposition process can interfere with the formation of pathways that are highly conductive in the crystal structure, resulting in a decrease in capacitance. This research reveals the notable impact of various growth methods on the capacitive properties of calcium carbonate crystals and emphasizes the potential of incorporating additives, such as kombucha, to improve their electrical conductivity.

The findings presented in Figure 6 have far-reaching implications for unconventional electronics. First, the remarkable rise in capacitance observed in fabrics infused with kombucha underscores the potential of utilizing natural additives to boost the electrical conductivity of crystal-based bioelectronic circuits. By incorporating these additives, it is possible to enhance the performance and functionality of these unconventional computing systems. Furthermore, the presence of negative capacitance in proteinoid composites indicates that the way in which calcium carbonate crystals are deposited and structured has a significant impact on their capacitive properties. Controlling the deposition process and optimizing the structural networking can lead to customized capacitance values, allowing for tailored information processing capabilities. Finally, the different crystal growth methods also offer valuable insights into the functionality and reliability of crystal bioelectronic circuits through their variations in capacitive tuning. Through careful selection of growth methods and additives, researchers can enhance the performance and stability of these circuits to suit specific applications. This knowledge has the potential to revolutionize the field of information processing by leveraging crystal-based materials, paving the way for innovative and unconventional computing systems.

### 3.4 Impedance and Phase Angle Analysis in Crystal Bioelectronic Circuits

Aside from characterizing capacitive properties, additional electrical profiling via impedance and phase measurement provides critical information on crystal circuit operation. Impedance (measured in ohms) includes total resistance opposing charge mobility through designed crystalline channels and logic gates-essentially an AC characterization of current flow restriction.

The impedance contributed by a capacitor in an electrical circuit is given by
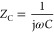
9where *Z*_C_ is the complex impedance of the capacitor (ohms), *j* is the imaginary unit, ω is the angular frequency (radians/s), and *C* is the capacitance (Farads).

This can be derived from
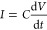
10
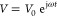
11
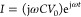
12where current *I* through a capacitor relates to the time derivative of voltage *V*.

Phase analysis deepens by evaluating the relative angular lag between cycling voltage and accompanying electrical fluctuations. Non-zero phase lags reveal the presence of other reactive contributors, specifically if capacitive or inductive elements disrupt current waveforms from remaining precisely synchronous to the ideal resistor setting. Multi-parameter electrical probing thus unveils internal propagation dynamics within interactive biotic-mineral arrays.

**Figure 7:**
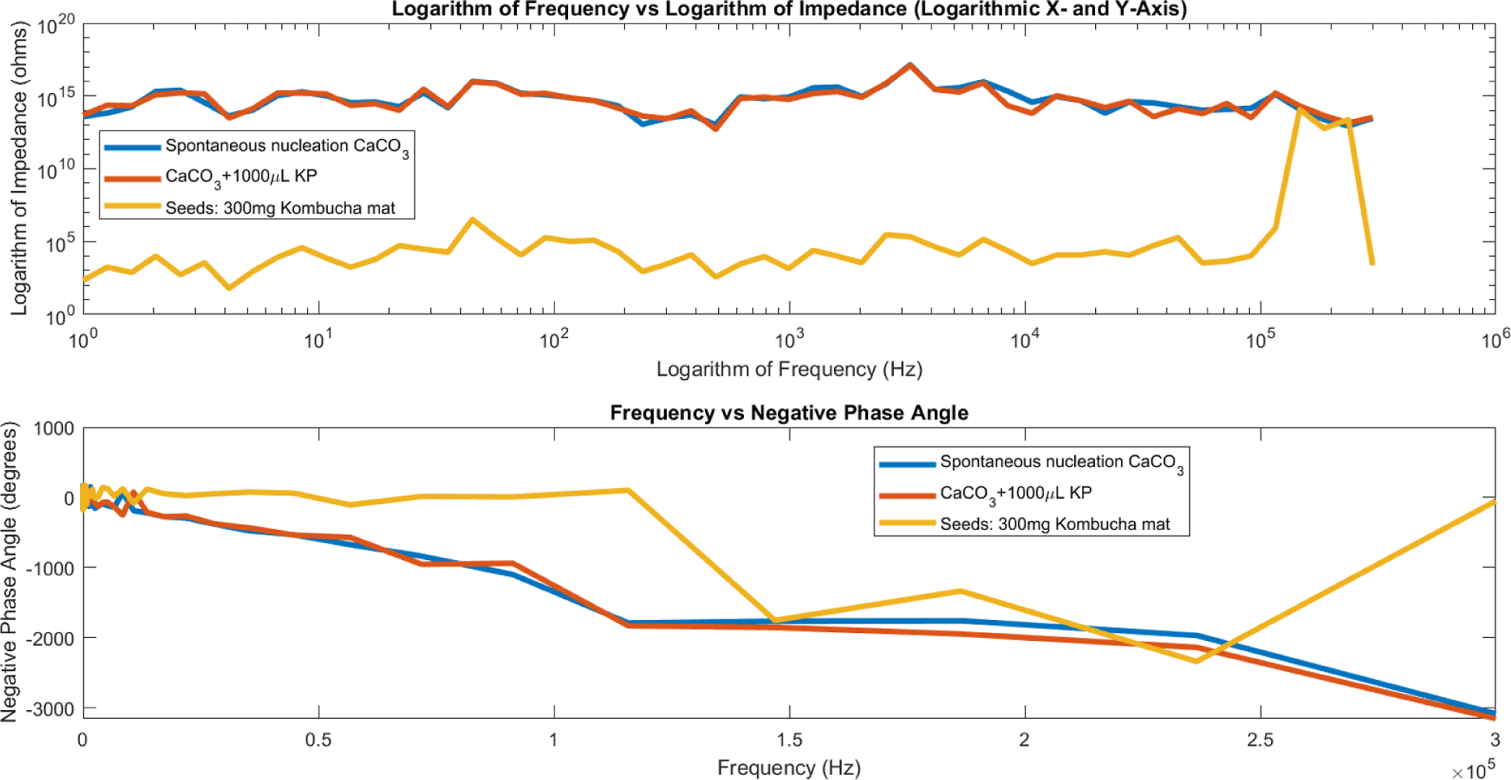
This graph compares the logarithm of impedance (*Z*) and phase angle for three different samples: CaCO_3_ spontaneous nucleation, CaCO_3_ + 1000 μL KP, and seeds: 300 mg kombucha mat. The top subplot plots the logarithm of impedance against the logarithm of frequency, providing insight into the relationship between impedance and frequency for each sample. The logarithmic frequency scale on the *x*-axis allows for the visualization of a wide range of frequencies, from 1 Hz to 1 MHz, enabling the observation of impedance behavior across several orders of magnitude. The negative phase angle is shown as a function of frequency in the bottom subplot, highlighting the phase mismatch between the voltage and current waveforms for the relevant samples. The following are the mean impedance (*Z*) values for each sample: sample 1:3.8935 × 10^15^ ohms for spontaneous nucleation CaCO_3_, 3.8935 × 10^15^ ohms for CaCO_3_ + 1000 μL KP, and 2.6291 × 10^12^ ohms. Furthermore, the mean values of negative phase angle for each sample are −295.18, −314.93, and −76.11° for CaCO_3_ spontaneous nucleation, CaCO_3_ + 1000 μL KP, and seeds: 300 mg kombucha mat, respectively.

The analysis of impedance and phase angle in the three distinct samples in this work offers useful insights into the electrical characteristics of crystal bioelectronic circuits (Figure 7). Plotting the logarithm of impedance versus the logarithm of frequency allows for the identification of unique patterns for each sample. The elevated mean impedance values found in sample 1 (spontaneous nucleation CaCO_3_) and sample 2 (CaCO_3_ + 1000 μL KP) indicate a significant resistance to the flow of electric current at different frequencies. On the other hand, the considerably reduced average impedance in sample 3 (seeds: 300 mg kombucha mat in a CaCO_3_ supersaturated solution) suggests a comparatively smoother passage of electric current. These findings emphasize the impact of various crystal growth mechanisms and additives on the electrical characteristics of crystal bioelectronic circuits.

The negative phase angle measurements provide insight into the phase shift between voltage and current waveforms in crystal bioelectronic circuits. The average negative phase angles obtained for each sample show the extent of phase disparity between the voltage and current signals. The elevated average negative phase angle values observed in sample 1 and sample 2 indicate a significant phase shift, suggesting a complicated circuit response and intricate interactions between electrical signals and crystal structures. Conversely, the reduced average negative phase angle seen in sample 3 suggests a less prominent phase shift, indicating a more direct electrical reaction when exposed to the kombucha-infused mat. The crystal growth can either facilitate the formation of interconnected tubule structures or impede the formation of structural networking.

### 3.5 Voronoi Diagram

The Voronoi diagram is a geometric construct utilized to partition a collection of points in space into distinct areas, each determined by its closest neighboring point. Within the realm of microspheres packing, the Voronoi diagram can unveil spatial connections within closely packed synthetic proteinoid spherules. The application of watershed segmentation on proteinoid distance map allows for the identification of boundaries that are equidistant from the nearest neighbors of each microsphere.

**Figure 8:**
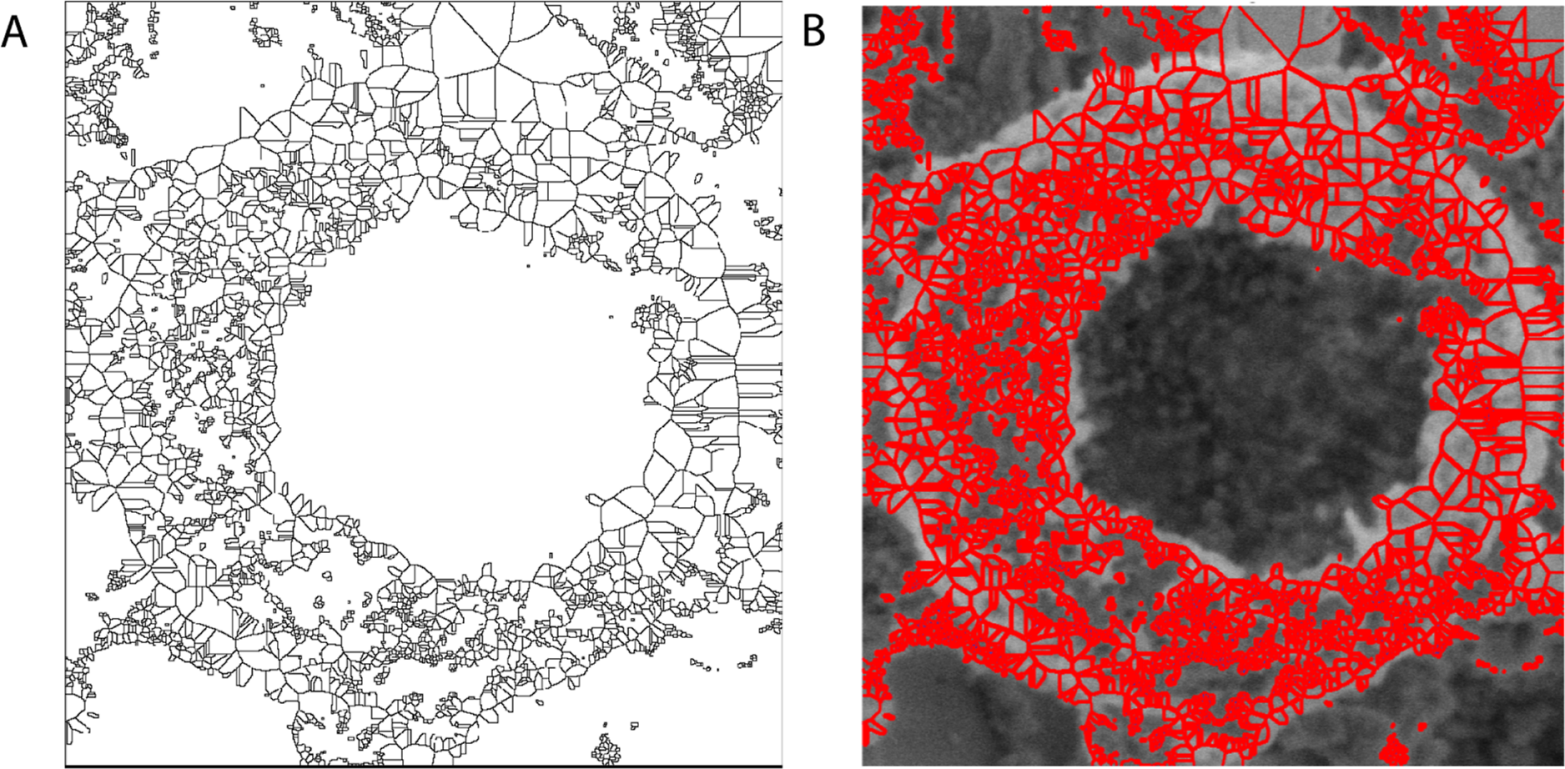
Microspheres formed during the spontaneous nucleation of calcium carbonate can be represented by a Voronoi diagram. (A) The diagram graphically illustrates the distribution and proximity of the microspheres, offering valuable insights into the nucleation process. (B) The identified edges are displayed in red, superimposed on the original image. Edge detection techniques facilitate the identification of the borders and changes between distinct areas or structures in the image, enabling a more thorough investigation of the microspheres.

This technique generates a mosaic map that illustrates the areas of effect or localized domains, demonstrating the relative arrangement of the microspheres within the overall packing ensemble. Figure 8 depicts the application of watershed segmentation on the microsphere packing, showcasing the resulting outcome. The mosaic map displays the specific areas of effect surrounding each microsphere, offering valuable insights into the spatial arrangement within the packing. Subsequently, it is possible to employ quantitative measurements on the individual Voronoi cells, such as computing the moments of the area distribution.

These metrics provide precise measurements of the variability among the several N-sided polygons that cover the intricate surface of the packing. If the metrics indicate mean values that are in close proximity to the ideals of hexagonal configurations, it suggests a collective cooperation toward achieving a uniform and densely packed arrangement. Conversely, if the measurements indicate uneven distribution, it implies that there is diversity within the arrangement of the microspheres, which may necessitate additional adjustments to their placements for optimal results. By comprehending packing symmetry, one can selectively stimulate particular Voronoi zones to activate localized sub-populations of the microspheres. Microscopic observations and measurements in specific places yield insights into the macroscopic responses and behaviors of the entire complex packing.

The MATLAB software was used to construct the Voronoi diagram. The Figure 8 underwent a conversion to grayscale, followed by thresholding to get a binary image. Finally, a distance transform was applied. Subsequently, the watershed segmentation technique was employed to acquire the Voronoi diagram, which accurately depicts the spatial arrangement of microspheres within the image. The area and perimeter of each Voronoi cell were calculated by doing measurements on the Voronoi diagram using the *regionprops* function. The average values of both the area and perimeter were calculated. In addition, the optimal values for a hexagonal layout were established, and the disparities between the average values and the hexagonal ideals were computed.

The Voronoi analysis of the microsphere packings provided significant insights on the spatial configuration and structure of the sample. The average area of the Voronoi cells was calculated to be (6.41 ± 0.05) × 10^3^ nm^2^, representing the typical size of the individual regions created by the microspheres. The average circumference of the cells was determined to be (4.48 ± 0.03) × 10^2^ nm, representing the combined length of all the boundaries in each region. In order to further assess the packing arrangement, the deviation between the average values of the area and perimeter and the optimal values for a hexagonal layout were computed. The discrepancy in the hexagonal area was calculated to be 6395.4 nm^2^, suggesting departures from the ideal hexagonal arrangement of the microspheres. Furthermore, a discrepancy of 419.9 nm was observed in relation to the hexagonal perimeter, indicating deviations in the total border length of the regions when compared to the anticipated hexagonal pattern. These findings offer valuable understanding of the general packing properties and uniformity within the examined system. The notable disparity between the hexagonal area and perimeter indicates a deviation from the perfect hexagonal arrangement, implying the existence of variances and possible anomalies in the positioning of the microspheres.

The mean free path (λ) is an important parameter that characterizes the average distance traveled by a particle before colliding. It reflects the average distance between subsequent collisions in the context of microspheres. To calculate the mean free path, we must first know the density (ρ = 2.66 g/cm^3^^76^) of microspheres in the supersaturated solution of the calcium carbonate and assume a certain particle distribution. In our investigation, we computed the number density based on the microsphere concentration, which was 3 × 10^–3^ M (moles per liter). We transformed this value to particles per cubic centimeter by multiplying it by Avogadro’s constant, which yields the number of particles per mole, and dividing by the molar volume of the microspheres. We estimated the mean free path assuming a random distribution of the microspheres. The formula was as follows
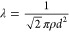
13where (λ) is the mean free path, (ρ) is the number density, and (*d*) is the diameter of the microspheres in nm. Substituting the calculated values, we obtained a mean free path of (1.87 × 10^–24^) nm.

### 3.6 Stimulus-Response Reshaping in Kombucha–Proteinoid Circuits

This section describes the electrical remodelling and structural reconfiguration that integrated kombucha–proteinoid networks undergo when driven by harmonically-varying low voltage sinusoids. The use of swept sinewave stimuli trains composite biofilms over time, achieving targeted transformation of initial quasi-periodic impulse patterns into reinforced signals phase-locked to conditioning waveforms. Constructive tuning leverages intrinsic sensory-motor plasticity beyond rudimentary modulation toward deep, gradual imprinting of periodicities matching stimulation parameters by incremental voltage-clamp training. If the fundamental frequency is defined as *f*, then the harmonics are given by
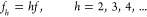
14where *h* is the harmonic number. For example, the second harmonic is 2*f*, third harmonic is 3*f*, etc. These overtones can be combined to construct more complex periodic signals. In the experiments, harmonics of *h* = 2, 3, 6, 10, 12, 15, 16 were applied. A simple sinusoidal wave function is defined as
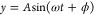
15where *A* is the amplitude, ω = 2π*f* is the angular frequency, *t* is time, and ϕ represents the phase offset. This can be readily extended to the harmonic frequencies by setting ω = 2π*hf*.

**Figure 9:**
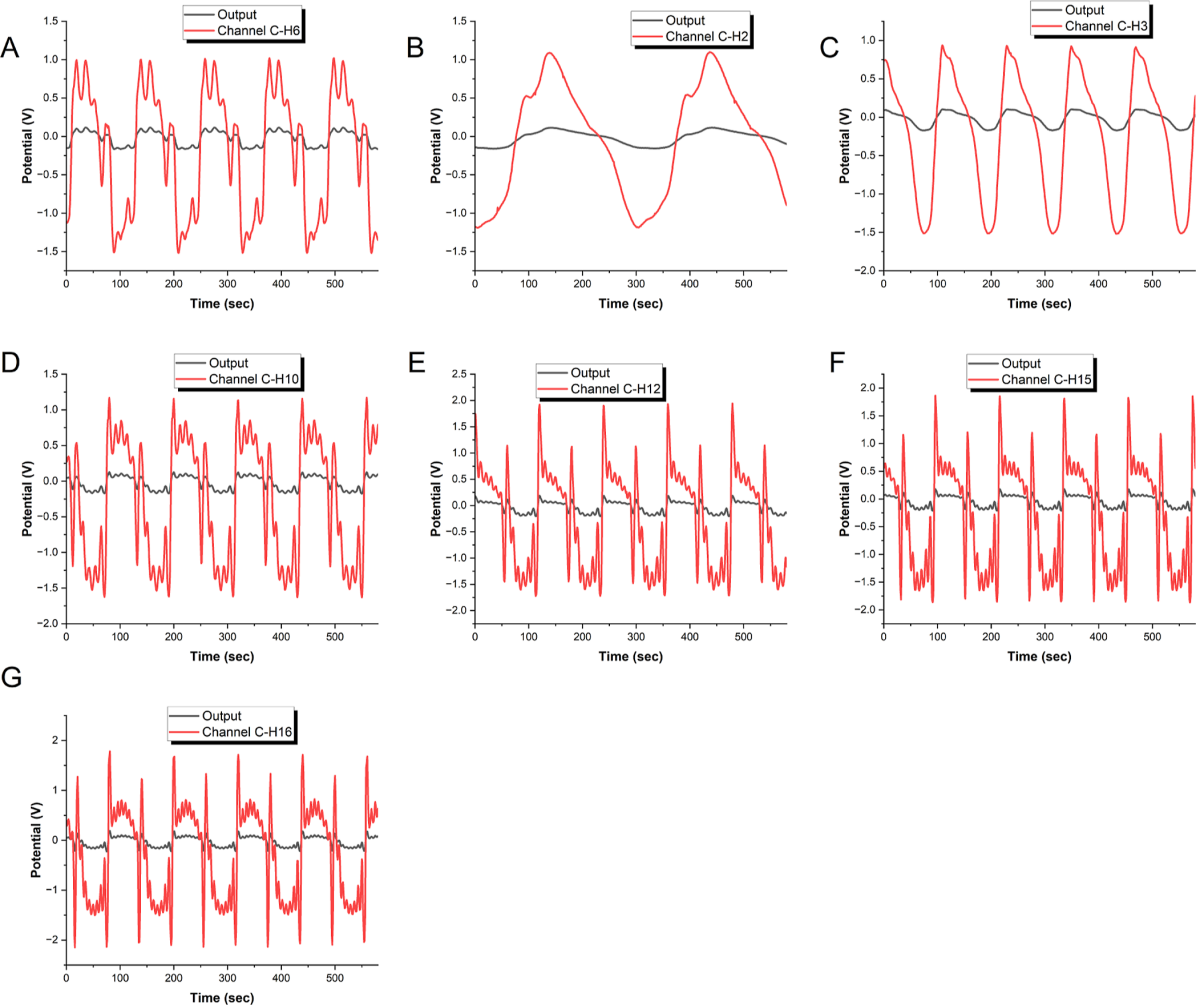
This figure depicts the interesting interplay between kombucha–proteinoids neuromorphic circuits immersed in a supersaturated calcium carbonate solution. When exposed to diverse sinusoidal input signals with harmonics (A) 2, (B) 3, (C) 6, (D) 10, (E) 12, (F) 15, and (G) 16, the neuromorphic circuits demonstrate amazing input–output behavior. Each subplot (A–G) corresponds to a specific harmonic frequency, showing the distinct response patterns. The input period is 1 min, and the input potential spans from 1 to −1 V. We get insights into the kombucha–proteinoid system’s computational and information processing capabilities by analyzing the output responses exhibited in this graph. It reveals the neuromorphic circuits’ ability to decipher and respond to specific input stimuli, underlining their potential for novel computer and cognitive information processing applications. This fascinating study adds to our understanding of the complex dynamics and emergent behaviors displayed by kombucha–proteinoids in reaction to their surroundings. It represents an interesting new direction for investigating the potential of bioelectronic systems for harvesting and altering information at the molecular level.

Figure 9 depicts the complex dynamics of kombucha–proteinoids proto-brains in a supersaturated calcium carbonate solution. The remarkable input–output behavior of the neuromorphic circuits is observed by subjecting them to a spectrum of sinusoidal input signals with harmonics 2, 3, 6, 10, 12, 15, and 16. The input period is precisely fixed at 1 min, with an input potential ranging from 1 to −1 V. We obtain vital insights into the electronic and information processing capabilities of the kombucha–proteinoid system by analyzing the graph. Notably, the pneuromorphic circuits have a remarkable ability to decode and respond to specific input stimuli, implying their prospective utility in unconventional computing and cognitive information processing applications.^77^

**Figure 10:**
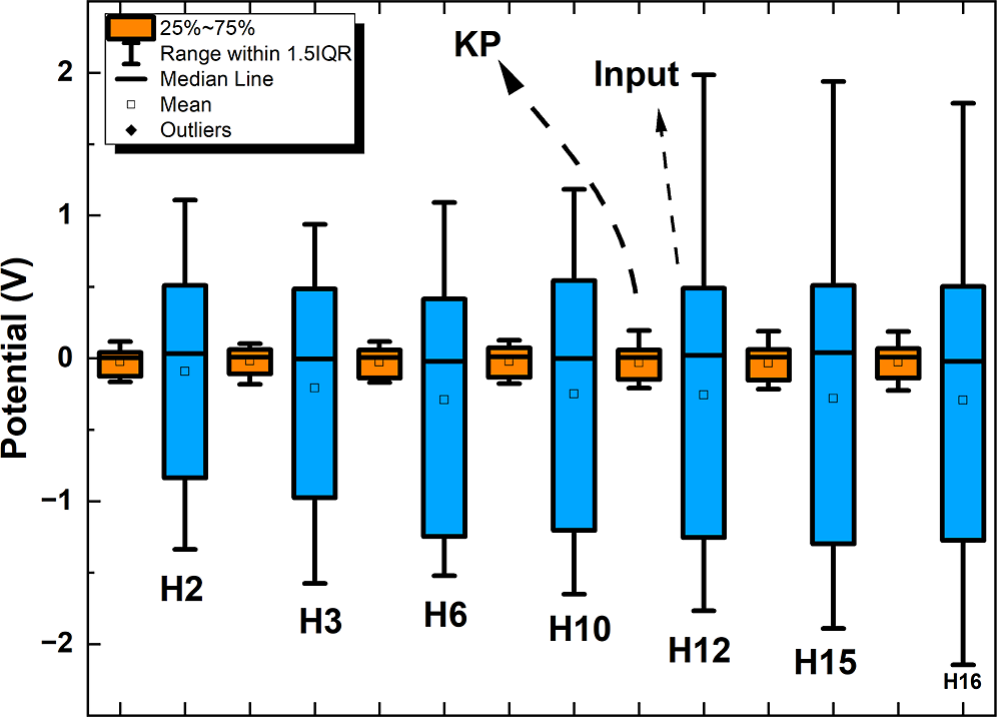
Figure presents the results of the statistical analysis performed on the Hamiltonians (Hamiltonians 2, 3, 6, 10, 12, 15, and 16) and the corresponding outputs of the KP-neuromorphic circuits system. The image displays bar charts that compare voltage distributions between sinewave stimuli and recordings from activated kombucha–proteinoid networks. The comparison is done over harmonic frequencies ranging from *h* = 2 to *h* = 16. The sinewave stimuli exhibit narrow and limited interquartile ranges, ranging from −0.15 to +0.15 V, with median values close to 0 V. Conversely, the results of the kombucha–proteinoid networks consistently demonstrate enhanced nonlinearity. The voltage distributions of the inputs exhibit a range of 1 V between the upper quartile (0.51 V) and the lower quartile (−1.29 V), with median fluctuations exceeding the magnitudes of the proteinoids. This variation in sensitivity indicates that as the inputs pass through the interacting synthetic tissue, they result in collective emergent outcomes. The voltage spreads are amplified as a result of the chaotic propagation throughout coupled proteinoid channels. In addition, the presence of higher harmonic orders seems to increase the spreads even more, suggesting the involvement of dynamics with higher dimensions. Although decoding the proteinoid outputs accurately may offer difficulties, the productive unpredictability that is naturally present in these oscillations has the potential to be utilized for training purposes. Utilizing the surplus and exploiting the inherent disorder in the system may provide opportunities for enhancing proteinoid structures for programmable bio-computation.

Figure 10 depicts the voltage distributions of the sinewave stimuli and the outputs produced from the stimulated kombucha–proteinoid networks. The bar charts depicted in this figure demonstrate that the sinewave stimuli display voltage ranges that are both narrow and limited, with the median values converging toward zero. However, the kombucha–proteinoid network outputs regularly exhibit enhanced nonlinearity. The voltage distributions of the proteinoid outputs exhibit a narrower dispersion, spanning a range of 0.3 V between the high and lower quartiles. The median values of the kombucha–proteinoid outputs exhibit both positive and negative fluctuations, surpassing the magnitudes of the stimuli.

This comparison indicates that when the inputs interact with the synthetic tissue of the proteinoid networks, they result in collective emergent outcomes. The disorderly transmission via coupled proteinoid channels seems to contribute to the increase in voltage spreads. In addition, the presence of higher harmonic frequencies appears to increase the range of values, suggesting the participation of dynamics with higher dimensions. This phenomenon emphasizes the unpredictable and divergent behavior of the proteinoid outputs, which may have consequences for utilizing inherent chaos and optimizing proteinoid structures for bio-computational purposes.

The *y*-axis denotes the “output potential,” which reflects the electrical response of the kombucha–proteinoid circuit to the applied harmonic signals. The potential is measured with voltage-sensitive probes at designated nodes within the biofilm network. The observed resonance phenomena result from the complex interaction between the applied harmonic stimuli and the inherent electrical characteristics of the kombucha–proteinoid network. The observed resonances indicate enhanced or reduced responses at particular frequencies, implying the existence of frequency-dependent amplification or damping mechanisms in the biofilm structure. To quantify these resonance effects, we define a frequency response function *H*(ω) as
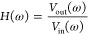
16where *V*_out_(ω) and *V*_in_(ω) are the Fourier transforms of the output and input voltages, respectively. The magnitude of *H*(ω) provides insight into the frequency-dependent gain of the system, while its phase reveals information about signal delays and potential phase-locking behaviors. The observed phase-locking and resonance effects in Figure 9, especially for harmonics 6, 12, and 16, indicate that the kombucha–proteinoid circuits demonstrate preferential responses to particular frequency ratios. This behavior may indicate emergent computational properties in bioelectronic systems, potentially facilitating frequency-based information processing or signal filtering capabilities. The resonance patterns and phase-locking behaviors illustrated in Figure 9, especially for harmonics 6, 12, and 16, indicate that kombucha–proteinoid circuits demonstrate emergent computational properties similar to those found in biological neural networks.^78^ Preferential responses to specific frequency ratios may suggest the existence of intrinsic oscillators within the biofilm structure, similar to those observed in cortical microcircuits.^79^ These oscillatory dynamics may allow bioelectronic systems to engage in frequency-based information processing, such as temporal coding^80^ and multiplexing.^81^ The observed nonlinear responses to harmonic stimuli resemble the frequency-following responses found in auditory systems,^82^ indicating potential applications in bioinspired signal processing and auditory computing.^83^ The capacity of kombucha–proteinoid networks to demonstrate advanced behaviors highlights their potential as a foundation for unconventional computing paradigms, connecting conventional electronic circuits with biological neural networks.^43,84^

**Figure 11:**
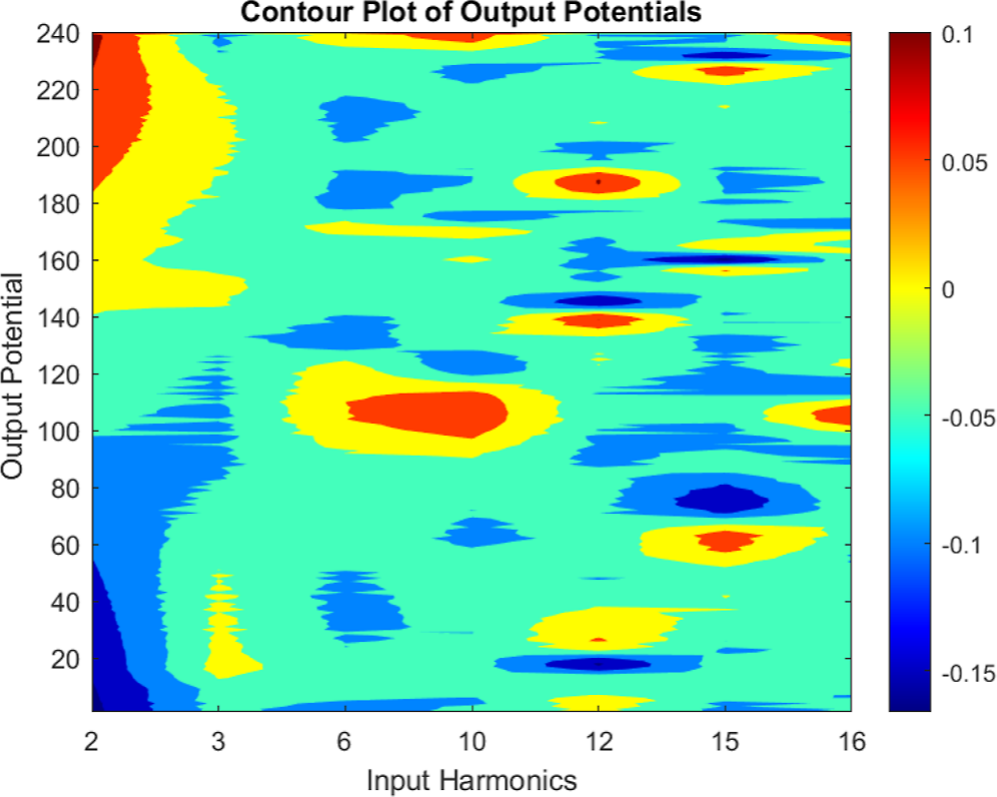
Contour map depicts the link between kombucha–proteinoid complexes output potentials and input harmonics (2, 3, 6, 10, 12, 15, and 16). Deeper outlines indicate larger potential values, whereas shallower contours imply lower potential values. The graphic shows that some input harmonic combinations result in larger output potentials, with a maximum value of 0.12 V. Other combinations, on the other hand, result in lower potentials, with a minimum value of −0.17 V. The average output potential across all combinations is −0.03 V, indicating a general trend toward lower potentials. This study sheds light on the effect of input harmonics on the reported kombucha–proteinoid dynamics.

The contour map in Figure 11 depicts the correlation between kombucha–proteinoid composite output potentials (shown on the *z*-axis) and input harmonics (represented on the *x*- and *y*-axes). The specific harmonics considered are 2, 3, 6, 10, 12, 15, and 16. The contour outlines depict the magnitude of the output potential values, where deeper contours correspond to higher potentials and shallower contours indicate lower potential values. The highest achievable output voltage of 0.12 V is obtained when the input harmonic combinations are situated within the most intense red contour in the diagram. Conversely, the shallowest blue contour corresponds to input combinations that yield a minimum output potential of −0.17 V. When taking into account all possible combinations of input harmonics, the mean output potential is −0.03 V. This indicates a general inclination of the neuromorphic circuits to generate comparatively low output potentials when exposed to the examined input harmonics. The contour map offers a comprehensive visualization of the influence of input harmonics on kombucha–proteinoid composite dynamics.

### 3.7 Entropy Rates and Algorithmic Complexity of Coupled pCa and pH Signals

The timecourse observations were analyzed to derive Kolmogorov complexity and entropy rates^85^ to evaluate the dynamics of the coupled calcium (pCa) and proton (pH) activity during crystallization. The Kolmogorov complexity (*K*) for both pCa and pH time series was calculated using a custom function kolmogorov() in MATLAB, which implements an approximation of Kolmogorov complexity based on the Lempel–Ziv compression algorithm. While the exact equation is complex, it can be conceptually represented as
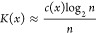
17where *c*(*x*) is the number of distinct patterns in the binary sequence *x*, and *n* is the length of the sequence. For the entropy rate calculations, we first computed the Shannon entropy (*H*) of the binary sequences derived from pCa and pH time series
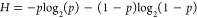
18where *p* is the mean of the binary sequence (proportion of 1’s). The entropy rate (ER) was then calculated by dividing the entropy by the average time step
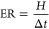
19where Δ*t* is the mean time difference between consecutive measurements.

The Kolmogorov complexity measures the least descriptive complexity, whereas the entropy rates measure predictability. When compared to the pH neuromorphic device, the pCa neuromorphic device had a larger Kolmogorov complexity (1.023 bits). This implies that changes in calcium ion concentrations necessitate a more detailed algorithmic description than changes in pH. pH dynamics, on the other hand, had marginally higher entropy, with a maximum rate of 1.000 vs 0.998 for pCa. Both measurements reached the theoretical maximum, indicating significant randomness in the entries’ sequential activity. Taken as a whole, the pH neuromorphic device exhibits greater unpredictability and complexity than pCa. The high entropy rates approaching 1 indicate the possibility of feedback processes causing chaos within the crystallization network. Although complex, tracing morphological developments and reaction cues may provide ways to exert control over emergent functionalities. Further quantification of computational capacities will decide the chances for specialized applications. Table 1 shows the results of calculating the Kolmogorov complexity and entropy rates for the combined pCa and pH neural signals. Mapping variations in precipitate morphology may offer ways to modulate the complex developing ionic waveforms and proton patterns. Nonetheless, complex signal signatures may offer specialized computational capacities by leveraging emergent growth behaviors.

**Table 1:** Kolmogorov Complexity and Entropy Rates Quantifying Dynamics and Unpredictability of Crystallization Network pCa and pH Activity^a^

neuromorphic device	Kolmogorov complexity (bits)	entropy rate
pCa	1.023	0.998
pH	0.456	1.000

a Entropy rates nearing theoretical maximum of 1 reflect considerable randomness within the coupled neuromorphic devices. The pCa neuromorphic device had a greater descriptive complexity, with a Kolmogorov complexity of 1.023 bits compared to the pH neuromorphic device’s 0.456 bits. The pH neuromorphic device, on the other hand, showed slightly greater unpredictability, with a peak entropy rate of 1.000 vs 0.998 for pCa.

**Figure 12:**
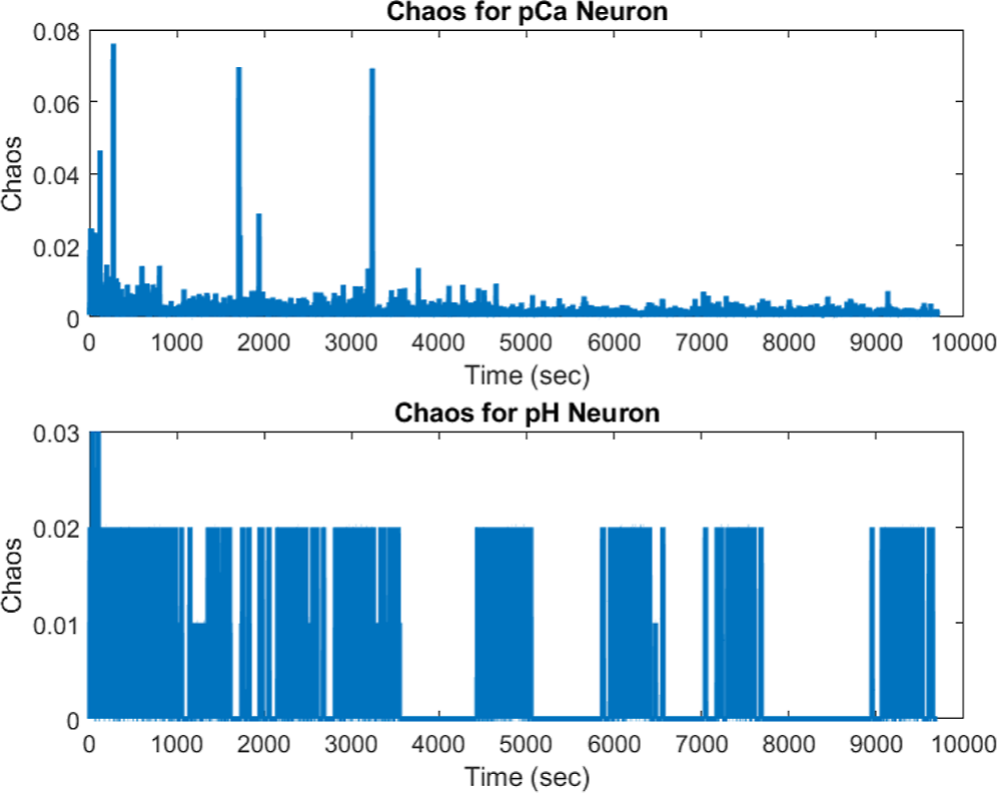
Crystallization network pCa and pH neuromorphic devices exhibit chaotic behavior. The upper panel depicts the chaotic trace for pCa activity across time, obtained by measuring the absolute second difference of recorded values. The chaotic pattern for pH is also seen in the lower plot. The oscillations reflect the unpredictability and randomness of calcium ion concentrations and proton activity.

The pCa and pH neuromorphic devices displayed signatures of chaos, as quantified by plots of the absolute second order difference over time (Figure 12). This transformation, calculated as
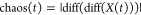
20where *X*(*t*) represents either pCa or pH at time *t*, reveals the unpredictable dynamical changes in the signals. As shown in Figure 12, considerable randomness is observed in both the calcium ion and proton patterns. The standard deviation over time quantifies the variance in the chaotic traces for both the pCa and pH neural signals. The standard deviation for the pCa chaotic pattern is 0.123498, showing a substantial spread around the mean that reflects the unpredictability of calcium ion fluctuations. The pH chaos trace, on the other hand, has a lower standard deviation of 0.042. In comparison to calcium ions, this implies greater constancy in the variability of proton activity. While both chaotic signatures exhibit substantial randomness, the fact that pCa has a greater standard deviation range suggests that its crystallization-induced dynamics may be more sensitive to exogenous control via disturbances. Controlling the larger chaos landscape may allow for directed functionality. Lower-dimensional pH chaotic traces, on the other hand, may bestow specialized computational capabilities within limited operational bounds.

The supplementary file details a method for quantifying calcium ion concentration. It includes a calibration curve for calcium ion-selective electrodes (Figure S1). This figure shows the correlation between the voltage and the log of calcium ion concentrations, from 3 to 4.75 mM. The supplementary file also includes the calibration equation (eq S1). It connects the observed potential to the molar concentration of calcium ions. It also examines the pH and pCa variations during seeded calcium carbonate crystallization, as shown in Figure S2. This figure shows the changes in pH and calcium ion activity over time. It reveals how calcium carbonate crystals form in supersaturated environments.

## 4 Discussion

This study shows the capacity to guide and program the growth dynamics of biotic–abiotic composites for unconventional computation. Our multi-modal experimental findings give several important insights. First, electron microscope images of complicated crystalline patterns show how kombucha–proteinoid interactions can control the branching self-assembly of calcium carbonate lattices, mixing soft hydrogel matrices with structured mineral deposits. Second, the electrical measurements show that conductive characteristics vary depending on growth methods, with spontaneous crystallization increasing resistance and seeded growth permitting conductive pathways. Finally, sweeping harmonic frequency inputs evoke unique signaling responses in the composite, resulting in complex oscillations and emerging logic processes. These findings show how the interaction of cellulose-producing bacteria, microsphere peptide assemblies, and propagating mineral formations results in specialized computational substrates. In the future, more investigation into the structure–function origins of observed dynamics, reinforced by modeling and characterization of morphological evolution across time, will help to enhance attempts to optimize, program, and scale guided living electronics for unconventional computation.

**Figure 13:**
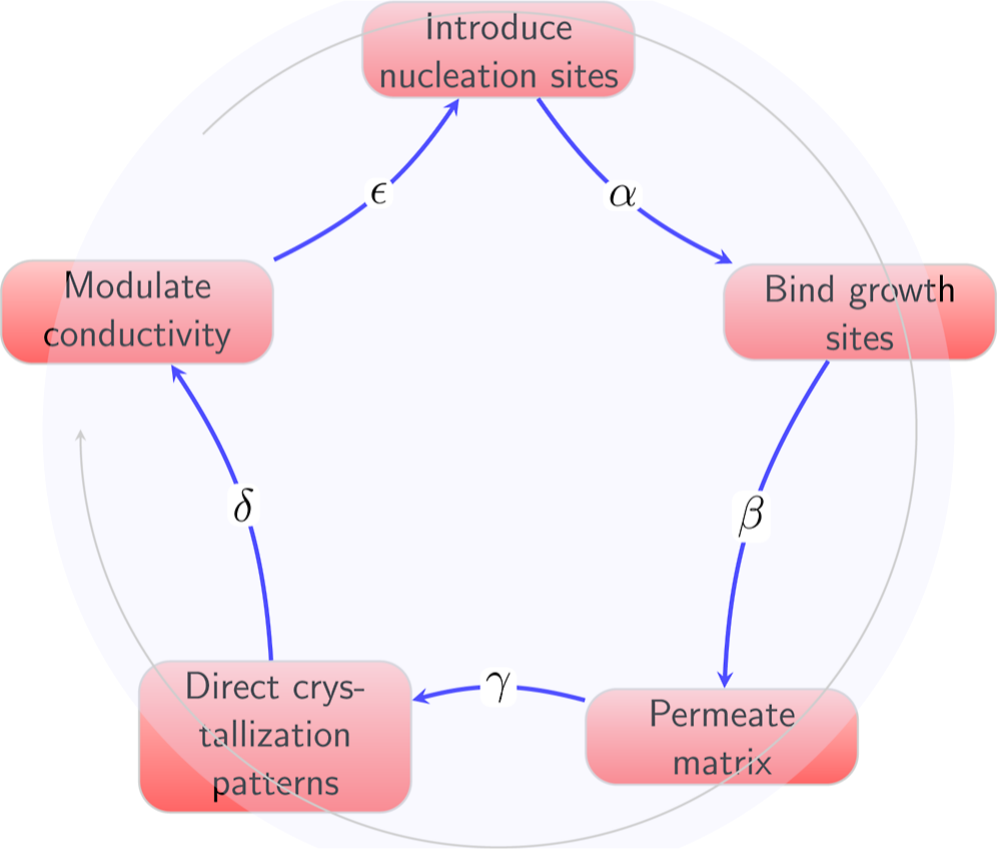
Proposed growth mechanism. The cultivated kombucha cellulose matrix (α) provides nucleation sites to guide crystallization. The added proteinoid microspheres (β) bind to propagating crystals to limit uncontrolled growth. The incubation period (γ) allows proteinoids to diffuse through the kombucha scaffold. Guided by cellulose-thermal proteins interactions, this directs the emerging crystal growth patterns (δ) and conductive paths formation, which emerges in modulated impedance levels (ϵ).

While more research is required, these findings give light on the underlying mechanism (Figure 13) of complex crystal growth dynamics and emergent computational capacities. Branching calcium carbonate structures are most likely formed by kombucha cellulose fibers and embedded microbial elements that direct and propagate mineralization outward into complex patterns led by cell wall surfaces. Meanwhile, proteinoid microspheres appear to inhibit uncontrolled crystalline overgrowth via binding interactions that may disrupt lattice propagation or modify energy barriers. In terms of electrical regulation, cellulose fibers and carbonate deposits can increase interior resistive paths, whereas protease inhibition protects conductive channels. It is hypothesized that the dynamic reconfiguration of propagation faces and dissolution patterns in the crystallizing composite architecture caused by oscillating inputs produces state-dependent reactions. The variations in resonant harmonics and frequency-selective outputs point to a complex interaction of capacitive and resistive effects inside the live electronic composite system as it grows and reconfigures.

The capacity to control and program crystal propagation paths brings up new opportunities for functional materials and computational tasks. Controlling the growth patterns and densities, for example, could allow for the recording of sophisticated multi-dimensional visual information within mineralized structures. Forming crystal growth in specific geometries may potentially enable shape-morphing architectures that twist, flex, or expand/contract when stimulated. Furthermore, the complex signaling dynamics seen in response to harmonic frequency sweeps suggest potential applications for arithmetic logic and modulatory filter operations. Through the control of crystallization and resonant interactions with different frequency bandwidths, the live electronic composites may perform low-power analogue data processing. In the future, further refining control over nucleation seeding, binding kinetics, propagator alignments, and morphological development will reveal the full scope of unconventional computing possibilities that can arise when exploiting these guided bio-abiotic growth dynamics. The integration of biological scaffold manufacturing and programmable abiotic replication at numerous interacting fronts provides a fertile ground for developing new kinds of functional computational materials.

On the experimental front, varied kombucha culture time frames, proteinoid polymer ratio, mineral precursors, and enlarged ranges of input harmonics are all possibilities. Sweeping a number of techniques with high-throughput strategies allows for quick mapping of dynamics and elucidation of optimal programming conditions. In addition to laboratory findings, physicochemical modeling of propagation reactions, binding phenomena, electron transfer, and crystallization processes might reveal mechanisms that allow for tailored responses. Simulations of morphological growth throughout time will also reveal connections between nano/micro-scale phenomena and macroscopic capacity. Evaluating other readouts such as changes in structural shape, mechanical characteristics, conductivity, and optical transmittance can reveal new functional modes beyond the electrical signaling first investigated. Finally, employing these smart materials to build specialized bio-electronic device designs offers up new possibilities. Kombucha–proteinoid-crystal composites, for example, may enable the printing of novel transistors and multiterminal logic gates for low-power biocomputation. They may also be useful as electrodes in cathode/anode bio-abiotic propagation circuits.

## 5 Conclusion

This work lays the framework for developing hybrid bio-abiotic seeds with directed growth dynamics and emergent computing capabilities. The multi-modal findings show how cooperative interactions between cellulose scaffolds created by microbes, thermal proteins assembly microspheres, and crystallizing minerals can control the formation of complicated architectures with specific electrical characteristics. Selective impedance modulation and complicated responses to external frequency modulation reveal the potential for information processing. These preliminary findings support important ideas for directing interaction biotic–abiotic growth systems. In the future, more research into binding processes, fabrication conditions, and dynamic structural reconfigurations will help to optimize morphological and computational control. Studies using high-throughput approaches to probe larger configuration spaces, physics-based modeling, and evaluations of alternative functional readouts will develop programmable living electronics. The possibilities range from bio-mimetic crystals that encode visual data to shape-changing actuators, arithmetic logic units that use crystallization density, and more. These early findings may eventually evolve into a rich platform for pioneering unconventional, growth-based computing systems with enhanced tuning of multi-component resonance. The guided processes emphasize the abundance of possibility at the interface of materials chemistry, synthetic biology, and morphological computation.

## Data Availability

This data is accessible via the online database Zenodo (https://zenodo.org/records/10300341).

## Supporting Information Available

Figures and equation (PDF)

## References

[ref1] Adamatzky A.; Bull L.; Costello B. D. L. Unconventional Computing 2007; Luniver Press, 2007.

[ref2] Adamatzky A. Advances in Unconventional Computing: Vol. 1: Theory; Springer, 2016; Vol. 22.

[ref3] Finocchio G.; Di Ventra M.; Camsari K. Y.; Everschor-Sitte K.; Khalili Amiri P.; Zeng Z. The promise of spintronics for unconventional computing. J. Magn. Magn. Mater. 2021, 521, 167506 10.1016/j.jmmm.2020.167506.

[ref4] Li S.; Kang W.; Zhang X.; Nie T.; Zhou Y.; Wang K. L.; Zhao W. Magnetic skyrmions for unconventional computing. Mater. Horiz. 2021, 8 (3), 854–868. 10.1039/d0mh01603a. 34821318

[ref5] Adamatzky A.; Akl S.; Burgin M.; Calude C. S.; Costa J. F.; Dehshibi M. M.; Gunji Y.-P.; Konkoli Z.; MacLennan B.; Marchal B.; et al. East-west paths to unconventional computing. Prog. Biophys. Mol. Biol. 2017, 131, 469–493. 10.1016/j.pbiomolbio.2017.08.004. 28818636

[ref6] Simpson M. L.; Cox C. D.; Peterson G. D.; Sayler G. S. Engineering in the biological substrate: Information processing in genetic circuits. Proc. IEEE 2004, 92 (5), 848–863. 10.1109/jproc.2004.826600.

[ref7] Hastings H. M. The substrate for biological information processing. In Molecular and Biological Physics of Living Systems; Springer, 1990; pp 111–122.

[ref8] Szaciłowski K. Digital information processing in molecular systems. Chem. Rev. 2008, 108 (9), 3481–3548. 10.1021/cr068403q. 18582117

[ref9] de la Escosura A. The informational substrate of chemical evolution: Implications for abiogenesis. Life 2019, 9 (3), 66 10.3390/life9030066. 31398942 PMC6789672

[ref10] Bourret R. B.; Stock A. M. Molecular information processing: lessons from bacterial chemotaxis. J. Biol. Chem. 2002, 277 (12), 9625–9628. 10.1074/jbc.r100066200. 11779877

[ref11] Benenson Y. Biomolecular computing systems: principles, progress and potential. Nat. Rev. Genet. 2012, 13 (7), 455–468. 10.1038/nrg3197. 22688678

[ref12] Wibral M.; Lizier J. T.; Priesemann V. Bits from brains for biologically inspired computing. Front. Robot. AI 2015, 2, 5 10.3389/frobt.2015.00005.

[ref13] Kitano H. Computational systems biology. Nature 2002, 420 (6912), 206–210. 10.1038/nature01254. 12432404

[ref14] Banga J. R. Optimization in computational systems biology. BMC Syst. Biol. 2008, 2 (1), 47 10.1186/1752-0509-2-47. 18507829 PMC2435524

[ref15] Păun G.; Pérez-Jiménez M. J. Membrane computing: brief introduction, recent results and applications. Biosystems 2006, 85 (1), 11–22. 10.1016/j.biosystems.2006.02.001. 16650521

[ref16] Eskov V. M.; Eskov V. V.; Vochmina J. V.; Gavrilenko T. V. The evolution of the chaotic dynamics of collective modes as a method for the behavioral description of living systems. Moscow Univ. Phys. Bull. 2016, 71, 143–154. 10.3103/S0027134916020053.

[ref17] Radzicki M. J. Institutional dynamics, deterministic chaos, and self-organizing systems. J. Econ. Issues 1990, 24 (1), 57–102. 10.1080/00213624.1990.11505001.

[ref18] Mella P.; et al. Combinatory systems and automata: Simulating self-organization and chaos in collective phenomena. Int. J. Knowl. Cult. Change Manag. 2007, 7 (2), 17–28. 10.18848/1447-9524/cgp/v07i02/50330.

[ref19] Allen P. M. Evolutionary complex systems: the self-organization of communities. In Complexity and Self-Organization in Social and Economic Systems: Proceedings of the International Conference on Complexity and Self-Organization in Social and Economic Systems Beijing, October 1994; Springer, 1997; pp 109–134.

[ref20] Abraham R. H. Dynamics and self-organization. In Self-Organizing Systems: The Emergence of Order; Springer, 1987; pp 599–613.

[ref21] Ditto W. L.; Sinha S. Exploiting chaos for applications. Chaos 2015, 25 (9), 097615 10.1063/1.4922976. 26428568

[ref22] Gentili P. L.; Giubila M. S.; Heron B. M. Processing binary and fuzzy logic by chaotic time series generated by a hydrodynamic photochemical oscillator. ChemPhysChem 2017, 18 (13), 1831–1841. 10.1002/cphc.201601443. 28160385

[ref23] Patten J.; Ishii H. Mechanical constraints as computational constraints in tabletop tangible interfaces. In Proceedings of the SIGCHI Conference on Human Factors in Computing Systems, 2007; pp 809–818.

[ref24] Schatz M. F.; Cicuta P.; Gordon V. D.; Pilizota T.; Rodenborn B.; Shattuck M. D.; Swinney H. L. Advancing access to cutting-edge tabletop science. Annu. Rev. Fluid. Mech. 2023, 55, 213–235. 10.1146/annurev-fluid-120720-025348.

[ref25] Proskurkin I. S.; Smelov P. S.; Vanag V. K. Experimental verification of an opto-chemical “neurocomputer. Phys. Chem. Chem. Phys. 2020, 22 (34), 19359–19367. 10.1039/d0cp01858a. 32822448

[ref26] Tomassoli L.; Silva-Dias L.; Dolnik M.; Epstein I. R.; Germani R.; Gentili P. L. Neuromorphic engineering in wetware: Discriminating acoustic frequencies through their effects on chemical waves. J. Phys. Chem. B 2024, 128 (5), 1241–1255. 10.1021/acs.jpcb.3c08429. 38285636

[ref27] Gorecki J.; Gizynski K.; Guzowski J.; Gorecka J. N.; Garstecki P.; Gruenert G.; Dittrich P. Chemical computing with reaction–diffusion processes. Philos. Trans. R. Soc., A 2015, 373, 20140219–20142015. 10.1098/rsta.2014.0219. 26078345

[ref28] D’Mello S. K.; Graesser A. Intelligent tutoring systems: How computers achieve learning gains that rival human tutors. In Handbook of Educational Psychology; Routledge, 2023; pp 603–629.

[ref29] Wang H.; Fu T.; Du Y.; Gao W.; Huang K.; Liu Z.; Chandak P.; Liu S.; Van Katwyk P.; Deac A.; et al. Scientific discovery in the age of artificial intelligence. Nature 2023, 620 (7972), 47–60. 10.1038/s41586-023-06221-2. 37532811

[ref30] Baracskai Z. Conventional non-computing and unconventional musical signal processing. In Unconventional Computing, Arts, Philosophy; World Scientific, 2023; pp 339–365.

[ref31] Zhu S.; Yu T.; Xu T.; Chen H.; Dustdar S.; Gigan S.; Gunduz D.; Hossain E.; Jin Y.; Lin F.; et al. Intelligent computing: the latest advances, challenges, and future. Intell. Comput. 2023, 2, 0006 10.34133/icomputing.0006.

[ref32] Qian L.; Winfree E.; Bruck J. Neural network computation with dna strand displacement cascades. Nature 2011, 475 (7356), 368–372. 10.1038/nature10262. 21776082

[ref33] Bray D. Protein molecules as computational elements in living cells. Nature 1995, 376 (6538), 307–312. 10.1038/376307a0. 7630396

[ref34] Păun G. Introduction to membrane computing. In Applications of Membrane Computing; Springer, 2006; pp 1–42. 10.1016/j.biosystems.2006.02.001 16650521

[ref35] Nakagaki T.; Yamada H.; Tóth Á. Maze-solving by an amoeboid organism. Nature 2000, 407 (6803), 470 10.1038/35035159. 11028990

[ref36] Tero A.; Takagi S.; Saigusa T.; Ito K.; Bebber D. P.; Fricker M. D.; Yumiki K.; Kobayashi R.; Nakagaki T. Rules for biologically inspired adaptive network design. Science 2010, 327 (5964), 439–442. 10.1126/science.1177894. 20093467

[ref37] Dorigo M.; Birattari M.; Stutzle T. Ant colony optimization. IEEE Comput. Intell. Mag. 2006, 1 (4), 28–39. 10.1109/ci-m.2006.248054.

[ref38] Fernando C. T.; Liekens A. M. L.; Bingle L. E. H.; Beck C.; Lenser T.; Stekel D. J.; Rowe J. E. Molecular circuits for associative learning in single-celled organisms. J. R. Soc., Interface 2009, 6 (34), 463–469. 10.1098/rsif.2008.0344. 18835803 PMC2582189

[ref39] Arnold F. H. Directed evolution: bringing new chemistry to life. Angew. Chem., Int. Ed. 2018, 57 (16), 4143–4148. 10.1002/anie.201708408. PMC5901037 29064156

[ref40] Pfeifer R.; Iida F.; Lungarella M. Cognition from the bottom up: on biological inspiration, body morphology, and soft materials. Trends Cognit. Sci. 2014, 18 (8), 404–413. 10.1016/j.tics.2014.04.004. 24839893

[ref41] Soloveichik D.; Seelig G.; Winfree E. Dna as a universal substrate for chemical kinetics. Proc. Natl. Acad. Sci. U.S.A. 2010, 107 (12), 5393–5398. 10.1073/pnas.0909380107. 20203007 PMC2851759

[ref42] Qian L.; Soloveichik D.; Winfree E. Efficient turing-universal computation with dna polymers. In DNA Computing and Molecular Programming: 16th International Conference, DNA 16, Hong Kong, China, June 14–17, 2010, Revised Selected Papers 16; Springer, 2011; pp 123–140.

[ref43] Adamatzky A. Physarum Machines: Computers from Slime Mould; World Scientific, 2010; Vol. 74.

[ref44] Adamatzky A. Hot ice computer. Phys. Lett. A 2009, 374 (2), 264–271. 10.1016/j.physleta.2009.10.072.

[ref45] Mougkogiannis P.; Adamatzky A. Memfractance of proteinoids. ACS Omega 2024, 9 (13), 15085–15100. 10.1021/acsomega.3c09330. 38585073 PMC10993267

[ref46] Hogg T.; Huberman B. A. Controlling smart matter. Smart Mater. Struct. 1998, 7 (1), R1–R14. 10.1088/0964-1726/7/1/001.

[ref47] Peter S.; Woitke L.; Dittrich P.; Ibrahim B. Computing all persistent subspaces of a reaction-diffusion system. Sci. Rep. 2023, 13 (1), 17169 10.1038/s41598-023-44244-x. 37821664 PMC10567720

[ref48] Grebenkov D. S. Diffusion-controlled reactions: an overview. Molecules 2023, 28 (22), 7570 10.3390/molecules28227570. 38005291 PMC10674959

[ref49] Weddig Karlsson A. Simulating nondiffusive dynamics in reaction-diffusion systems. Master’s Thesis, Chalmers University of Technology, 2023.

[ref50] Adamatzky A. Towards fungal computer andrew adamatzky. Fungal Machines: Sensing and Computing with Fungi; Springer Nature, 2023; Vol. 47, p 245.

[ref51] Martínez G. J.; Adamatzky A.; Schroeder M. J. Unconventional computing art in cellular automata. In Unconventional Computing, Arts, Philosophy; World Scientific, 2023; pp 291–303.

[ref52] Mougkogiannis P.; Adamatzky A. Proto-neurons from abiotic polypeptides. Encyclopedia 2024, 4 (1), 512–543. 10.3390/encyclopedia4010034.

[ref53] Adamatzky A. Electrical potential spiking of kombucha zoogleal mats: A symbiotic community of bacteria and yeasts. Bioelectricity 2023, 5 (2), 99–108. 10.1089/bioe.2022.0030.

[ref54] Viswan N. A.; Bhalla U. S. Understanding molecular signaling cascades in neural disease using multi-resolution models. Curr. Opin. Neurobiol. 2023, 83, 102808 10.1016/j.conb.2023.102808. 37972535

[ref55] Roberts N.; Raeisi Kheirabadi N.; Tsompanas M.-A.; Chiolerio A.; Crepaldi M.; Adamatzky A. Logical circuits in colloids. 2023, arXiv preprint arXiv:2307.02664 10.1098/rsos.231939 PMC11285612 39076794

[ref56] Owolabi K. M.; Agarwal R. P.; Pindza E.; Bernstein S.; Osman M. S. Complex turing patterns in chaotic dynamics of autocatalytic reactions with the caputo fractional derivative. Neural Comput. Appl. 2023, 35, 11309–11335. 10.1007/s00521-023-08298-2.

[ref57] Awad A.; Pang W.; Lusseau D.; Coghill G. M. A survey on physarum polycephalum intelligent foraging behaviour and bio-inspired applications. Artif. Intell. Rev. 2023, 56 (1), 1–26. 10.1007/s10462-021-10112-1.

[ref58] Liu J.; Fu Y.; Li Y.; Zhou H. A novel improved slime mould algorithm for engineering design. Soft Comput. 2023, 27 (17), 12181–12210. 10.1007/s00500-023-08430-3.

[ref59] Gharehchopogh F. S.; Ucan A.; Ibrikci T.; Arasteh B.; Isik G. Slime mould algorithm: A comprehensive survey of its variants and applications. Arch. Comput. Methods Eng. 2023, 30 (4), 2683–2723. 10.1007/s11831-023-09883-3. 36685136 PMC9838547

[ref60] Vaishampayan V.; Kapoor A.; Gumfekar S. P. Enhancement in the limit of detection of lab-on-chip microfluidic devices using functional nanomaterials. Can. J. Chem. Eng. 2023, 101, 5208–5221. 10.1002/cjce.24915.

[ref61] Dkhar D. S.; Kumari R.; Malode S. J.; Shetti N. P.; Chandra P. Integrated lab-on-a-chip devices: Fabrication methodologies, transduction system for sensing purposes. J. Pharm. Biomed. Anal. 2023, 223, 115120 10.1016/j.jpba.2022.115120. 36343538

[ref62] Mencattini A.; Rizzuto V.; Antonelli G.; Di Giuseppe D.; D’Orazio M.; Filippi J.; Comes M. C.; Casti P.; Vives Corrons J. L.; Garcia-Bravo M.; et al. Machine learning microfluidic based platform: Integration of lab-on-chip devices and data analysis algorithms for red blood cell plasticity evaluation in pyruvate kinase disease monitoring. Sens. Actuators, A 2023, 351, 114187 10.1016/j.sna.2023.114187.

[ref63] Atkinson N.; Morhart T. A.; Wells G.; Flaman G. T.; Petro E.; Read S.; Rosendahl S. M.; Burgess I. J.; Achenbach S. Microfabrication process development for a polymer-based lab-on-chip concept applied in attenuated total reflection fourier transform infrared spectroelectrochemistry. Sensors 2023, 23 (14), 6251 10.3390/s23146251. 37514546 PMC10383751

[ref64] Bakhtiari Ramezani S.; Sommers A.; Kumar Manchukonda H.; Rahimi S.; Amin A. Machine learning algorithms in quantum computing: A survey. In 2020 International Joint Conference on Neural Networks (IJCNN); IEEE, 2020; pp 1–8.

[ref65] Adamatzky A. A brief history of liquid computers. Philos. Trans. R. Soc., B 2019, 374 (1774), 20180372 10.1098/rstb.2018.0372. PMC6553589 31006363

[ref66] Eiben A. E.; Kernbach S.; Haasdijk E. Embodied artificial evolution: Artificial evolutionary systems in the 21st century. Evol. Intell. 2012, 5, 261–272. 10.1007/s12065-012-0071-x. 23144668 PMC3490067

[ref67] Fox S. W.; Nakashima T. The assembly and properties of protobiological structures: The beginnings of cellular peptide synthesis. BioSystems 1980, 12 (3–4), 155–166. 10.1016/0303-2647(80)90013-1. 7397322

[ref68] Nguyen V. T.; Flanagan B.; Gidley M. J.; Dykes G. A. Characterization of cellulose production by a gluconacetobacter xylinus strain from kombucha. Curr. Microbiol. 2008, 57, 449–453. 10.1007/s00284-008-9228-3. 18704575

[ref69] Mayne R.; Adamatzky A. Slime mould foraging behaviour as optically coupled logical operations. Int. J. Gen. Syst. 2015, 44 (3), 305–313. 10.1080/03081079.2014.997528.

[ref70] Tanaka H. Viscoelastic phase separation in biological cells. Commun. Phys. 2022, 5 (1), 167 10.1038/s42005-022-00947-7.

[ref71] Saffioti N. A.; Cavalcanti-Adam E. A.; Pallarola D. Biosensors for studies on adhesion-mediated cellular responses to their microenvironment. Front. Bioeng. Biotechnol. 2020, 8, 597950 10.3389/fbioe.2020.597950. 33262979 PMC7685988

[ref72] Hannah S.; Al-Hatmi M.; Gray L.; Corrigan D. K. Low-cost, thin-film, mass-manufacturable carbon electrodes for detection of the neurotransmitter dopamine. Bioelectrochemistry 2020, 133, 107480 10.1016/j.bioelechem.2020.107480. 32045862

[ref73] Adamatzky A.; Harding S.; Erokhin V.; Mayne R.; Gizzie N.; Baluška F.; Mancuso S.; Sirakoulis G. C. Computers from plants we never made: Speculations. In Inspired by Nature: Essays Presented to Julian F. Miller on the Occasion of his 60th Birthday; Springer, 2017; pp 357–387.

[ref74] Mougkogiannis P.; Phillips N.; Adamatzky A. Transfer functions of proteinoid microspheres. Biosystems 2023, 227–228, 104892 10.1016/j.biosystems.2023.104892. 37076037

[ref75] Ershov M.; Liu H. C.; Li L.; Buchanan M.; Wasilewski Z. R.; Jonscher A. K. Negative capacitance effect in semiconductor devices. IEEE Trans. Electron. Dev. 1998, 45 (10), 2196–2206. 10.1109/16.725254.

[ref76] Kamhi S. R. On the structure of vaterite caco3. Acta Crystallogr. 1963, 16 (8), 770–772. 10.1107/s0365110x63002000.

[ref77] Piccinini G.; Scarantino A. Information processing, computation, and cognition. J. Biol. Phys. 2011, 37, 1–38. 10.1007/s10867-010-9195-3. 22210958 PMC3006465

[ref78] Izhikevich E. M. Dynamical Systems in Neuroscience; MIT Press, 2007.

[ref79] Buzsáki G.; Draguhn A. Neuronal oscillations in cortical networks. Science 2004, 304 (5679), 1926–1929. 10.1126/science.1099745. 15218136

[ref80] Panzeri S.; Brunel N.; Logothetis N. K.; Kayser C. Sensory neural codes using multiplexed temporal scales. Trends Neurosci. 2010, 33 (3), 111–120. 10.1016/j.tins.2009.12.001. 20045201

[ref81] Akam T.; Kullmann D. M. Oscillatory multiplexing of population codes for selective communication in the mammalian brain. Nat. Rev. Neurosci. 2014, 15 (2), 111–122. 10.1038/nrn3668. 24434912 PMC4724886

[ref82] Skoe E.; Kraus N. Auditory brain stem response to complex sounds: a tutorial. Ear Hear. 2010, 31 (3), 302–324. 10.1097/aud.0b013e3181cdb272. 20084007 PMC2868335

[ref83] Lyon R. F. Human and Machine Hearing: Extracting Meaning from Sound; Cambridge University Press, 2017.

[ref84] Kirchhoff M. D.; Froese T. Where there is life there is mind: In support of a strong life-mind continuity thesis. Entropy 2017, 19 (4), 169 10.3390/e19040169.

[ref85] Morzy M.; Kajdanowicz T.; Kazienko P.; et al. On measuring the complexity of networks: Kolmogorov complexity versus entropy. Complexity 2017, 2017, 1–12. 10.1155/2017/3250301.

