# Supplementary material for: Kombucha–Proteinoid Crystal Bioelectric Circuits
Source: ACS Omega. 2024 Oct 28;9(45):45386–401. doi: 10.1021/acsomega.4c07319 (PMC11561624; doi:10.1021/acsomega.4c07319)
Supplement: Supplementary file 1 — ao4c07319_si_001.pdf [file ao4c07319_si_001.pdf]

Supporting Information for: Kombucha–Proteinoid Crystal Bioelectric Circuits

Panagiotis Mougkogiannis*[1], Anna Nikolaidou[1,2], and Andrew Adamatzky[1]

[1]Unconventional Computing Laboratory, University of the West of England, Coldharbour Ln, Stoke Gifford, Bristol, BS16 1QY, UK
[2]School of Architecture and Environment, University of the West of England, Coldharbour Ln, Stoke Gifford, Bristol, BS16 1QY, UK

Supporting Information

This document contains additional information to support the main manuscript. It includes a detailed description of the calcium ion concentration quantification method, along with supplementary figures and equations.

Calcium Ion Concentration Quantification

Calcium ion concentrations were quantified using specialized Ca^{2+}–selective electrodes that were calibrated over a range from 3 mM to 4.75 mM, as detailed in the calibration curve provided in Figure S1.

$$E = 1.229 + 0.219 \ln[\mathrm{Ca}^{2+}] \quad (1)$$

Equation S1 links the observed potential (E) in volts to the molar concentration of calcium ions Ca^{2+}, (moles per litre). The calibration curve converts electrode voltage data to precisely calculate calcium ion concentration in test liquids or hydrogel samples during repeated measurements. These calcium ion–selective electrodes and calibration process are trustworthy tools for accurate calcium quantification in crystallising Kombucha–proteinoid composite systems due to their high R^2 value and wide concentration range.

*Corresponding author: Panagiotis.Mougkogiannis@uwe.ac.uk

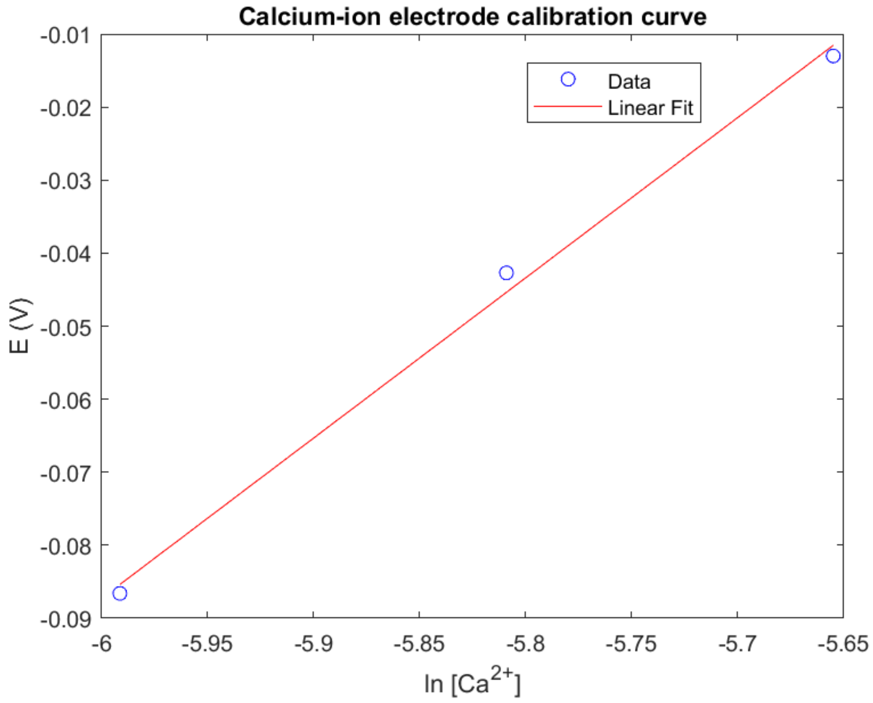

Figure 1: Calibration curve for calcium ion–selective electrodes. The calibration plot shows the relationship between the measured voltage (E) of the ion–selective electrode and the logarithm of calcium ion concentration [Ca^{2+}] spanning from 3 mM to 4.75 mM. A linear regression fit of the data yields an R^2 value of 0.996, indicating a strong correlation.

As mineral nucleation and growth occur on seed particles under supersaturated conditions, the surrounding solution's pH and calcium ion activity can alter. The pH rapidly decreased from 8.73 to 8.49 within the first 4,600 seconds of commencing seeded calcium carbonate crystallisation, as illustrated in Figure S2. This transitory dip may come from the release of protons (H^+) during the early stages of directed calcite formation on seed surfaces, before declining into a slower crystal development phase. As the reactions progress towards equilibrium, such proton release occurs as inorganic carbonate species transform into crystalline calcium carbonate lattices ($CaCO_3$). After much of fast hydrolysis and crystal stacking has occurred, the pH returns to initial values after 79,900 seconds. The linked pCa changes reflect the concurrent depletion of free calcium ions from solution as they

combine into the solid mineral phase. As a result, the instantaneous pH drop and long–term pCa decrease provide insights into the kinetics of seeded calcium carbonate crystalline formation under the investigated supersaturated conditions.

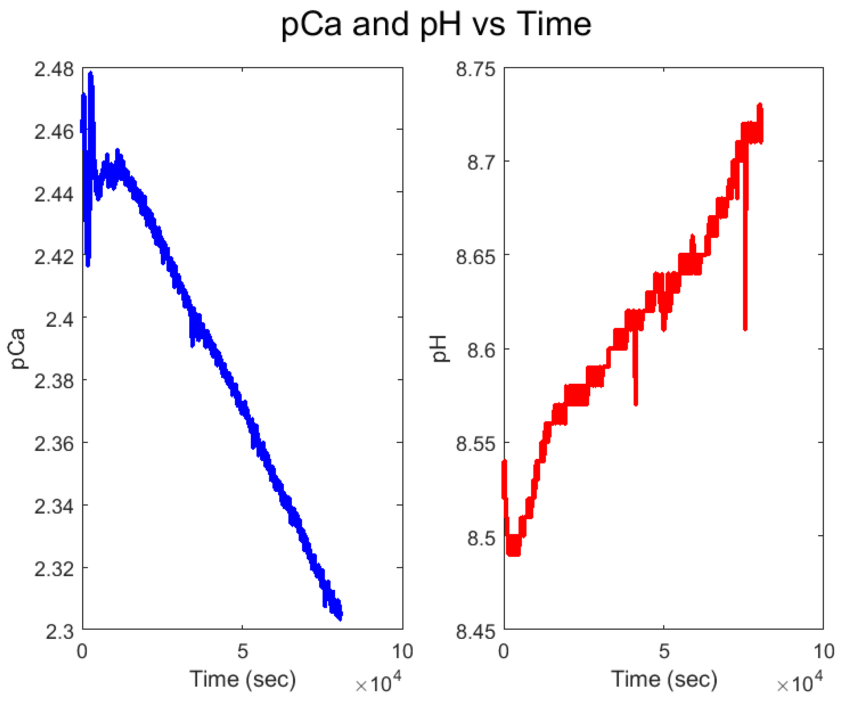

Figure 2: Changes in pH and pCa over time during seeded calcium carbonate crystallisation. The studies took place in supersaturated $CaCO_3$ solutions with initial calcium and carbonate concentrations of 3 mM. Over the first 4,600 seconds, the pH rapidly lowers from 8.73 to 8.49. This is most likely due to proton release during initial mineral nucleation and crystallisation on seed particles. The pH rebounds to 8.73 after this point up to 79,900 seconds as crystal growth rates slow. Meanwhile, pCa values begin at 2.45, indicating high calcium ion activity, and steadily decrease to 2.30 by 80,578 seconds. This shows that free calcium ions are being depleted from solution as they form mineral crystals on seed surfaces. The combined variations in pH and calcium activity provide insight into the precipitation kinetics of calcium carbonates during seeded crystallisation under supersaturated conditions.
